# Probe-based bacterial single-cell RNA sequencing predicts toxin regulation

**DOI:** 10.1038/s41564-023-01348-4

**Published:** 2023-04-03

**Authors:** Ryan McNulty, Duluxan Sritharan, Seong Ho Pahng, Jeffrey P. Meisch, Shichen Liu, Melanie A. Brennan, Gerda Saxer, Sahand Hormoz, Adam Z. Rosenthal

**Affiliations:** 1 IFF Health and Biosciences, Wilmington, DE USA; 2 Lewis-Sigler Institute for Integrative Genomics, Princeton University, Princeton, NJ USA; 3 Harvard Graduate Program in Biophysics, Harvard University, Cambridge, MA USA; 4 Department of Data Science, Dana-Farber Cancer Institute, Boston, MA USA; 5 Department of Chemistry and Chemical Biology, Harvard University, Cambridge, MA USA; 6 Department of Systems Biology, Harvard Medical School, Boston, MA USA; 7 Broad Institute of MIT and Harvard, Cambridge, MA USA; 8 Department of Microbiology and Immunology, University of North Carolina, Chapel Hill, NC USA

**Keywords:** Bacteriology, Cellular noise, Pathogens

## Abstract

Clonal bacterial populations rely on transcriptional variation across individual cells to produce specialized states that increase fitness. Understanding all cell states requires studying isogenic bacterial populations at the single-cell level. Here we developed probe-based bacterial sequencing (ProBac-seq), a method that uses libraries of DNA probes and an existing commercial microfluidic platform to conduct bacterial single-cell RNA sequencing. We sequenced the transcriptome of thousands of individual bacterial cells per experiment, detecting several hundred transcripts per cell on average. Applied to *Bacillus subtilis* and *Escherichia coli*, ProBac-seq correctly identifies known cell states and uncovers previously unreported transcriptional heterogeneity. In the context of bacterial pathogenesis, application of the approach to *Clostridium perfringens* reveals heterogeneous expression of toxin by a subpopulation that can be controlled by acetate, a short-chain fatty acid highly prevalent in the gut. Overall, ProBac-seq can be used to uncover heterogeneity in isogenic microbial populations and identify perturbations that affect pathogenicity.

## Abstract (web summary)

ProBac-seq is a method that uses libraries of DNA probes and commercial microfluidics for single-cell RNA-seq, leveraged here to show heterogeneous gene expression in clonal bacterial cultures including variable toxin expression in an agricultural strain of *Clostridium*.

## Main

Bacterial traits such as competence, sporulation and motility have been shown to be both heterogeneously utilized in populations and tightly controlled at the transcriptional level, emphasizing the need for single-cell transcriptional analyses^1–3^. However, tools for single-cell RNA sequencing (scRNA-seq) of bacterial populations remain limited due to several substantial technical challenges. First, total bacterial messenger RNA (mRNA) abundance is two orders of magnitude lower than that of eukaryotes, with a single bacterial cell containing approximately 10^3^–10^4^ transcripts during exponential growth^4^. Second, transcriptional turnover is much faster in bacteria, with an mRNA half-life on the scale of minutes, compared with hours in eukaryotes^4^. Third, bacterial transcripts do not intrinsically include a 3′ poly-adenosine tail and, therefore, mRNA cannot easily be tagged and selectively enriched against ribosomal RNA, which makes up more than 90% of the bacterial transcriptome^4^. Lastly, accessing mRNA requires cell permeabilization, which is difficult in bacteria due to the diversity in membrane structures and variability of the peptidoglycan layer.

We reasoned that a method combining the advantages of microfluidic single-cell barcoding in droplets with the ability to tag transcripts using in situ hybridization of oligonucleotide probes could overcome these challenges and offer advantages over alternative approaches^5–7^. Here we present a method called probe-based bacterial sequencing (ProBac-seq) for prokaryotic scRNA-seq, which uses a commercial, benchtop microfluidic device (Chromium Controller from 10X Genomics) and custom single-stranded DNA probe libraries to resolve the mRNA profile of thousands of bacterial cells.

## Results

### Probe design and library generation

**Fig. 1:**
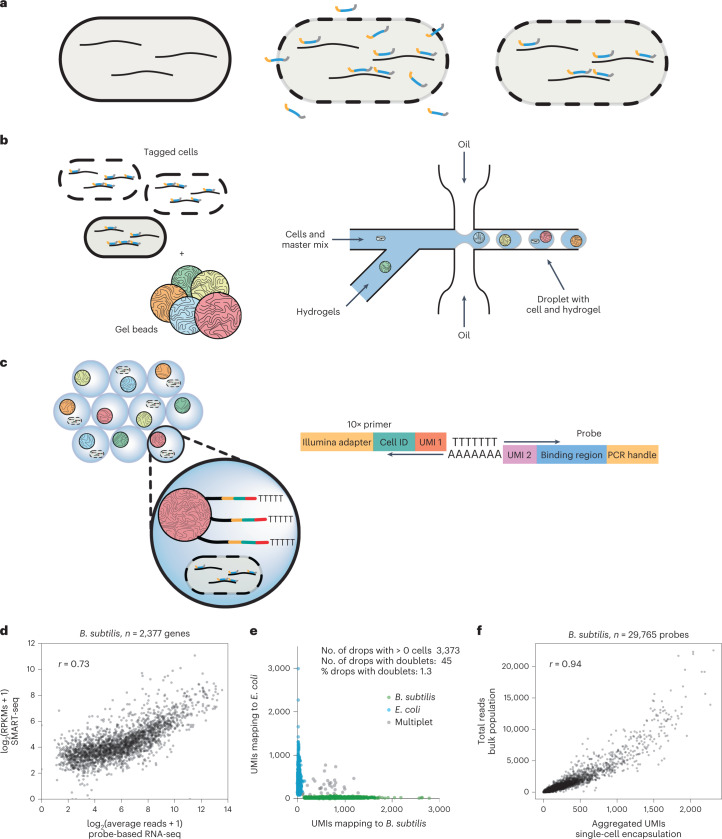
Microfluidic probe-based scRNA-seq method and validation. **a**, Cells were fixed and permeabilized to allow the penetration of thousands of unique, genome-specific oligonucleotide probes. Hybridized probes retrofitted transcripts with a poly-A tail and UMI, whereas unhybridized probes were washed away. **b**, Permeabilized cells with hybridized probes were flowed through a commercial microfluidic device that encapsulates single cells into droplets containing barcoded primers with poly-A capture sequence conjugated to a hydrogel microsphere and PCR reagents. **c**, Final droplets contain one or fewer cells and one hydrogel with a unique cell barcode. Barcoded cDNA was generated from the mRNA:probe hybridized complex via in-droplet PCR. Droplets were then broken and the pooled cDNA amplified further before sequencing. Single-cell transcriptomes were resolved, clustered and visualized. **d**, Transcriptome quantification by hybridization of a probe library followed by PCR correlates (Pearson’s correlation coefficient, *r* = 0.73) to traditional, bulk RNA-seq method (SMART-seq stranded kit, Takara) involving random priming of hexamers followed by reverse transcription (RT) and incorporation of template switching oligo. **e**, Species mixture (‘barnyard’) plot demonstrates that single cells of different bacterial species can be resolved by barcode after microfluidic encapsulation. **f**, Aggregated probe-based signal from thousands of single cells is well correlated (Pearson’s correlation coefficient, *r* = 0.94) to the average probe-based signal obtained from the bulk population (pre-encapsulation). RPKM, reads per kilobase of exon per million reads mapped.

To leverage existing microfluidic single-cell sequencing platforms, we devised a method whereby individual transcripts are tagged with DNA probes. This approach requires generating large oligonucleotide libraries that are complementary to the protein-coding sequences within a genome (Supplementary Fig. 1). Large transcriptome-wide oligonucleotide libraries have been extensively used for bulk transcriptomics in microarrays and, more recently, to selectively capture mRNA from an infection model that includes mammalian and bacterial transcripts^8^. In our design, multiple DNA regions of 50 base pairs (bp) were chosen from each open-reading frame based on uniqueness as determined by UPS2 software^9^ or based on published oligonucleotide arrays^10,11^. These sequences then served as the target regions of single-stranded DNA probes, which were designed to hybridize to the corresponding mRNA by sequence complementarity. Probes also contained a 5′ polymerase chain reaction (PCR) handle for library generation, a unique molecular identifier (UMI) and a 3′ poly-A tail (A_30_) for retrofitting prokaryotic transcripts to the 10X Genomics Single Cell 3′ system (Fig. 1a). Multiple probes (complementary to different regions) were designed for each gene to enhance transcript-capture efficiency and decrease noise caused by poor hybridization and/or insufficient amplification of any given probe. Complete species libraries contained 29,765 probes for *Bacillus subtilis*, 21,527 probes for *Escherichia* *coli* and 11,723 probes for *Clostridium perfringens* and targeted 2,959 (*B. subtilis*) 4,181 (*E. coli*) and 3,189 (*C. perfringens*) genes, respectively. Libraries were ordered at subfemtomole quantities from Twist Biosciences (a one-time cost of US$0.05–0.15 per probe) and amplified by rolling circle amplification^12^ to obtain a sufficient amount (0.25 mg = 10.25 nmol per library or approximately 0.35 pmol of each probe) for scRNA-seq experiments (Methods, Supplementary Tables 1–3 and Supplementary Figs. 1 and 2). Probe libraries were completed by addition of randomized 12 bp UMI sequences and a poly-A tail and purified. Read coverage across completed probe libraries was well approximated by a log normal distribution (Supplementary Fig. 1e). The rolling circle amplification approach permits re-amplifying probe sets for unlimited subsequent experiments without the need to re-order probes, at an upfront cost of under US$0.01 per cell (Supplementary Table 4).

Before microfluidic encapsulation, bacteria were fixed in 1% paraformaldehyde to preserve transcripts and permeabilized by mild lysozyme treatment. Permeabilized bacteria were then incubated with the corresponding DNA probe library. Non-hybridized probes were removed with repeated washes (Fig. 1, Methods). Next, bacteria were run through a 10X Chromium Controller, where DNA probes were captured and barcoded in a manner analogous to the barcoding of the transcriptome of eukaryotic cells (Fig. 1b–d). The resulting libraries were sequenced, preprocessed with custom scripts extracting the target sequence, cell barcode and UMI (https://gitlab.com/hormozlab/bacteria_scrnaseq) and analysed with the standard CellRanger pipeline and the Seurat analysis package (Methods)^13,14^.

### Bacterial scRNA-seq validation

To determine if suspended probe libraries can report on the transcriptional state of cells, we split a culture of *B. subtili*s cells in a late exponential state into two aliquots of approximately 10^8^ formaldehyde-fixed cells each. One aliquot was processed using a traditional RNA-seq protocol (Methods), whereas the second sample was processed by in situ hybridization with the *B. subtilis* probe set. After hybridization and washing, the probes that remained bound were amplified by PCR and processed into Illumina libraries. The output from each method was compared. A probe-based library prepared with hybridization and wash conditions at 50 °C gave results similar to traditional RNA-seq (Fig. 1e; Pearson’s *r* = 0.73). Repeating in *E. coli* with its respective probe set, we observed a Pearson correlation of 0.77 (Supplementary Fig. 19). These values are comparable to the correlation between RNA-seq and microarray experiments (*r* = 0.75–0.80)^15,16^.

Next, we tested if single-cell transcriptomes can be captured by encapsulating individual bacteria using the 10X Chromium Controller. *E. coli* and *B. subtilis* cells were independently fixed and pretreated with probes corresponding to their respective genomes. The prepared bacterial samples were subsequently mixed and loaded onto a microfluidic chip along with a PCR master mix containing a DNA primer designed to amplify the back-end PCR handle built into the probes (Supplementary Fig. 1, Methods). This custom mix replaced the standard cDNA reagents supplied by 10X Genomics as the aqueous phase within the droplets. Sequenced libraries from this experiment demonstrate that the microfluidic platform can successfully segregate and barcode individual microbial cells (Fig. 1f). We observed a corrected multiplet rate of 2.8% for 3,373 captured cells, consistent with the expected rate of successful single-cell encapsulation based on the specifications of the 10X microfluidic system (expected 2.4% multiplet events per 3,000 recovered cells).

To assess whether the signal from individually encapsulated cells provides a representative readout of the transcriptomic state of the population, we compared the output produced by capturing probes from a bulk sample of cells (≅10^8^ cells) to the signal obtained by summing the probe counts over thousands of individually tagged cells (aggregated UMIs). The abundances across probes between the two samples were highly correlated (*r* = 0.94, Fig. 1g), confirming the signal from single cells provides a good representation of transcriptional states.

### ProBac-seq identifies heterogeneity in *B. subtilis*

We performed ProBac-seq on *B. subtilis* grown to late exponential phase in M9 minimal media supplemented with malate. In total, 2,784 cells were captured as determined by unique barcodes at an average depth of 1,073 reads per cell. We detected a median of 325 mRNA transcripts per cell (approximately 10–20% of the total mRNA pool^4,17^) corresponding to a median of 241 genes per cell. UMIs per probe ranged from 0 to 45 for any given gene in any given cell. The most highly detected probes targeted genes encoding ribosomal proteins (*rpsG*, *rpsH*, *rpsR*, *rpsM*, *rpsS*, *rplL*, *rplD*, *rplO*, *rplV*), translation elongation machinery (*fusA*, *tufA*, *map*) and transcriptional machinery (*rpoA*).

**Fig. 2:**
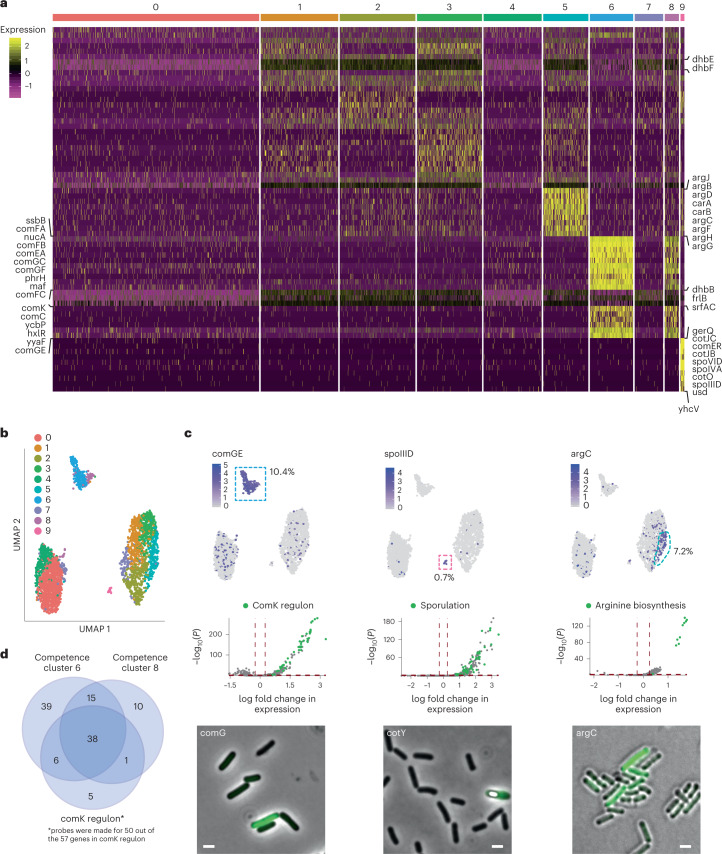
ProBac-seq analysis reveals distinct transcriptional states in *B. subtilis*. **a**, Heatmap of marker gene expression (*z*-score of log-transformed values) from 2,784 individual *B. subtilis* cells organized into 10 clusters. **b**, UMAP two-dimensional representation of the 10 cell clusters reveals four highly distinct transcriptomic signatures. **c**, Top: single-cell expression of key marker genes for competence (comGE, clusters 6 and 8), sporulation (spoIIID, cluster 9) and arginine synthesis (argC, cluster 5) are highlighted on the UMAP (left to right). Cells within the highlighted cluster are boxed by dashed lines. Middle: volcano plots of genes expressed in at least 25% of cells in the corresponding clusters, with genes from the respective processes highlighted in green. *P* values correspond to two-sided Wilcoxon rank-sum test with Bonferroni correction. Lower: presence of each heterogeneous marker in the population as confirmed by fluorescent promoter-reporter constructs (P_*comGE*_-YFP, P_*cotY*_-YFP and P_*argC*_-YFP, respectively). Representative images. A phase bright spore can be seen in the cotY-expressing cell. Scale bars, 1 µm. Microscope images were collected from at least ten fields with three biological replicates per reported strain to quantify phenotypes **d**, Of all comK regulon genes probed, 90% (45 out of 50) were significantly differentially upregulated in cell clusters 6 and 8 (Bonferroni-corrected *P* value ≤ 0.05 for each gene). See Supplementary Table 11 for information on comK regulon genes probed.

Next, we resolved distinct cellular states by dimensional reduction of single-cell expression vectors followed by graph-based clustering and analysis of differential gene expression (DGE) using the Seurat package and two-sided Wilcoxon rank-sum test with Bonferroni correction^13^. For *B. subtilis* in M9 minimal media in late log growth, we resolved four major transcriptomic states comprising 10 cell clusters that capture subtler signatures in gene expression (Fig. 2a,b, Supplementary Fig. 3 and Supplementary Table 6). Algorithmic grouping of cell-expression profiles was robust to the clustering parameters (Methods). As expected for *B. subtilis* at late log phase in minimal media, we observed a subpopulation of cells (clusters 6 and 8) with signatures of genetic competence. Cells in these two clusters differentially overexpress the competence master-regulator, comK, compared with the rest of the population (log_2_fold change (FC) = 2.37, adjusted *P* value = 4 × 10^−179^) and multiple genes within and downstream of competence operons (*comC*, *G*, *E* and *F* operons, *coiA*, *dprA*, *sbB*, *nucA*, *nin*, *clpC* and *recA*). The fraction of competent cells in the population was measured by fluorescence microscopy using a fluorescent promoter-reporter of *comGE* (Methods, Fig. 2c left panel and Supplementary Fig. 4a) and determined to be approximately 10.4% (interquartile range (IQR) outlier detection; Methods) similar to the fraction of the population comprising clusters 6 and 8 (9.3%, Supplementary Table 5). In total, we found that 45 of the 50 comK regulon genes that were targeted with probes were differentially overexpressed in clusters 6 and 8 (adjusted *P* values < 0.05; Fig. 2d and Supplementary Table 6). Our results agree with numerous studies identifying the competence regulon in *B. subtilis*^18,19^ and the percentage of cells displaying natural competence falls within previous observations for similar media (3–10%)^20,21^.

Unexpectedly, for these growth conditions we observed a small subpopulation of cells within the sample (cluster 9, 19 cells, 0.7% of the population) with a transcriptomic signature indicative of sporulation. In these cells, we observed significant upregulation of transcripts corresponding to sigma factors associated with sporulation, *sigF* (log_2_FC = 1.2, adjusted *P* value = 4 × 10^−41^) and *sigG* (log_2_FC = 2.2, adjusted *P* value = 2 × 10^−63^), and genes in the *ger, cot* and *spoIVF* operons. Gene set enrichment analysis (GSEA) on all significantly upregulated genes in cluster 9 using ontological classes from the Gene Ontology Consortium^22–24^ reveals a 4.3-fold enrichment of genes involved in sporulation (false discovery rate (FDR) = 1 × 10^−16^, Fisher’s exact test) and spore germination (fold enrichment = 8.1, FDR = 1.7 × 10^−3^). This finding highlights ProBac-seq’s ability to detect rare and unexpected cell states. To confirm this subpopulation, we created a promoter-reporter for *cotY*, a spore coat gene expressed in the sporulating mother cell. Fluorescence observation identifies the presence of *cotY* expression and spores in a similar percentage of cells as recovered by scRNA-seq (0.5–1%) (Fig. 2c middle panel, Supplementary Fig. 4d).

The largest grouping of *B. subtilis* cells (comprising clusters 1, 2, 3, 5 and part of 7) accounted for ~45% of the population and was characterized primarily by the upregulation of the *dhb* operon (log_2_FCs > 0.25, adjusted *P* values < 2 × 10^−5^). Genes *dhbA, dhbB, dhbC, dhbE* and *dhbF* encode the five enzymes implicated in the biosynthesis of bacillibactin—a catecholic siderophore that is produced and secreted in response to intracellular iron deprivation. Relatedly, *yusV*, which encodes an ABC transporter of bacillibactin, was also upregulated in these cells. Cells within cluster 5 are distinguished from the rest of the subpopulation by expression of genes related to arginine biosynthesis via ornithine. In total, 10 of 14 genes associated with the cellular process were significantly upregulated in this cluster alone (gene set fold enrichment = 19.35, FDR = 3 × 10^−8^). A fluorescent promoter-reporter for *argC* confirms heterogeneous expression of arginine genes with approximately 2.1% of cells in the high-argC expressing tail of the distribution, compared with 7.2% of cells in this state as determined by scRNA-seq (Fig. 2c right panel, Methods, Supplementary Fig. 4b and Supplementary Table 5).

Prokaryotes often express genes with related functions as operons; polycistronic transcripts under the control of a single promoter. In many cases, we observed genes of the same operon overexpressed within the same cluster of cells, serving as an internal control for our RNA capture and clustering methods. To further explore the ability of ProBac-seq to resolve operons agnostically, pairwise comparisons of gene expression levels across cells were computed using Spearman distance as a measure of covariance. Pairwise distances were then related by agglomerative hierarchical clustering (average linkage) to identify sets of covarying genes. Compared to scrambled controls, genes with significant covariance (empirical *P* value < 3 × 10^−5^) often recapitulated known operons in *B. subtilis* (Supplementary Fig. 5 and Supplementary Table 7), providing strong evidence that probes capture true transcriptomic signatures across individual cells.

Taken together, our platform for single-cell sequencing of individual bacterium correctly recapitulates the known cellular states of *B. subtilis* at the expected population fractions and identifies previously unreported cellular states and was able to uncover features of the underlying genomic architecture.

### Expression heterogeneity in a clonal *E. coli* population

**Fig. 3:**
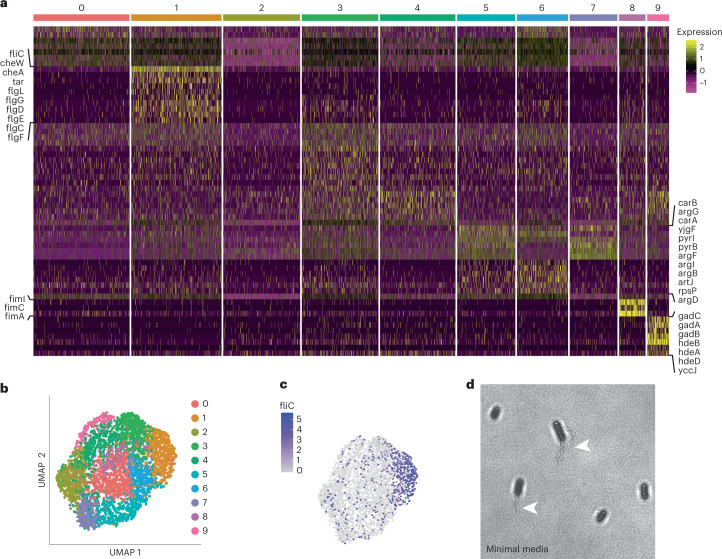
Heterogeneous gene expression in *E. coli* grown in minimal medium. **a**, Heatmap of marker gene expression (*z*-score of log-transformed values) from 3,315 individual *E. coli* cells grown aerobically in minimal M9 media organized into 10 clusters. **b**, UMAP two-dimensional representation of the 10 cell clusters from aerobic M9 culture conditions. **c**, Flagellar components are heterogeneously expressed in aerobic M9 media, a key flagellar gene (flagellin, *fliC*), is preferentially expressed by cells in cluster 1. **d**, The heterogeneous presence of flagella in the population is confirmed by flagellar staining.

With a validated method for identifying cellular states in *B. subtilis*, we used ProBac-seq to characterize transcriptional heterogeneity in *E. coli* MG1655 that was grown in M9 minimal media and chemically fixed at an optical density OD_600_ = 0.5 (mid-log phase). We resolved 3,315 cells at an average depth of 1,070 reads per cell, detecting a median of 263 transcripts per cell representing a median of 165 distinct genes. Cells were partitioned into nine groups by graph-based clustering of their gene expression profiles (Fig. 3a,b, Supplementary Fig. 6 and Supplementary Table 6). As with *B. subtilis*, we observe that certain clusters can be assigned to specific biological processes by DGE and that cluster determination is robust to the choice of clustering parameters (Methods). For example, cells in cluster 1 (482 cells, 14.5% of population) uniquely upregulate genes implicated in cell motility, including those encoding chemotaxis signalling proteins (*cheA, cheW, tar*; gene set fold enrichment > 100, FDR = 3.1 × 10^−2^) and structural flagella components (*fliC, flgL, flgG, flgD, flgE, flgC, flgF*; gene set enrichment = 86.08, FDR = 2.6 × 10^−6^). This list includes genes regulated by class 1, 2 and 3 flagella promoters; three distinct regulons that control the sequential expression of flagella genes including the master-regulator *flhDC*, components of the membrane-associated basal body and chemotaxis proteins, respectively^25^. To confirm the presence of a motile subpopulation of cells, we stained cells grown under the same conditions using Remel Flagella Stain and visualized them through phase microscopy (Fig. 3d). This revealed that approximately 36% of cells had assembled flagella, which was a greater proportion than the 14.5% observed in the scRNA-seq cluster 1. A possible explanation for this discrepancy is that some flagellated cells may have already exited the transcriptional state, but still retained the translated protein product.

Cells within clusters 5, 6 and 7 demonstrate differential usage of carbamoyl phosphate within the culture. Clusters 5 and 7 upregulate *carA* and *carB* (log_2_FC > 0.87, adjusted *P* values < 1 × 10^−14^), which encode the subunits of carbamoyl phosphate synthetase, which catalyses the conversion of bicarbonate to carbamoyl phosphate for de novo biosynthesis of both uridine-5′-monophosphate and arginine. Both clusters, arranged adjacently on the uniform manifold approximation and projection (UMAP), also upregulate *pyrB, pyrC* and *pyrI*, (log_2_FC > 0.4, adjusted *P* values < 6 × 10^−^^3^), three enzymes involved in the first two steps of uridine-5′-monophosphate biosynthesis, implying a commitment of the cells towards pyrimidine biosynthesis. Alternatively, cells in cluster 6 appear to shuttle carbamoyl phosphate into the arginine biosynthesis pathway as implied by the upregulation of *argB, argD, argF, argG, argI* (log_2_FC > 0.55, adjusted *P* values < 1 × 10^−^^20^). GSEA reinforces this observation, finding a significant enrichment of genes implicated in arginine biosynthesis via ornithine in these cells (gene set fold enrichment = 26.77, FDR = 2.02 × 10^−^^3^).

Cells in cluster 8 (141 cells, 4.3% of the population) exhibit significant upregulation of *fimI, fimC* and *fimA* (log_2_FC > 2.4, adjusted *P* value < 1 × 10^−^^166^). The downstream genes of the fim operon encode for components of type 1 pili, which are associated with biofilm formation and pathogenicity^26–28^ and are known to be expressed heterogeneously. Taken together, our observations in *E. coli* and *B. subtilis* suggest that extensive phenotypic heterogeneity is a general feature of bacteria, with processes such as arginine metabolism often confined to specialist cells. Additionally, genes associated with virulence segregate to specific cell populations, suggesting that specialized cell types may be involved in pathogenicity.

### Toxin-expression heterogeneity in *C. perfringens*

After validating ProBac-seq on model organisms, we sought to examine whether we could identify distinct subpopulations in a bona fide pathogen. We focused on toxin production in *C. perfringens*, a Gram-positive, spore-forming bacterium that is an important pathogen of humans and livestock and exhibits one of the fastest cell division times reported in literature (6.3 min)^4,29^. The virulence factor in type A *C. perfringens* responsible for necrotic enteritis disease is a secreted *β*-barrel pore-forming toxin, NetB. In poultry, necrotic enteritis caused by *C. perfringens* Avian type A strain is a major challenge to antibiotic-free farming and causes annual worldwide losses of approximately US$6 billion^30^.

**Fig. 4:**
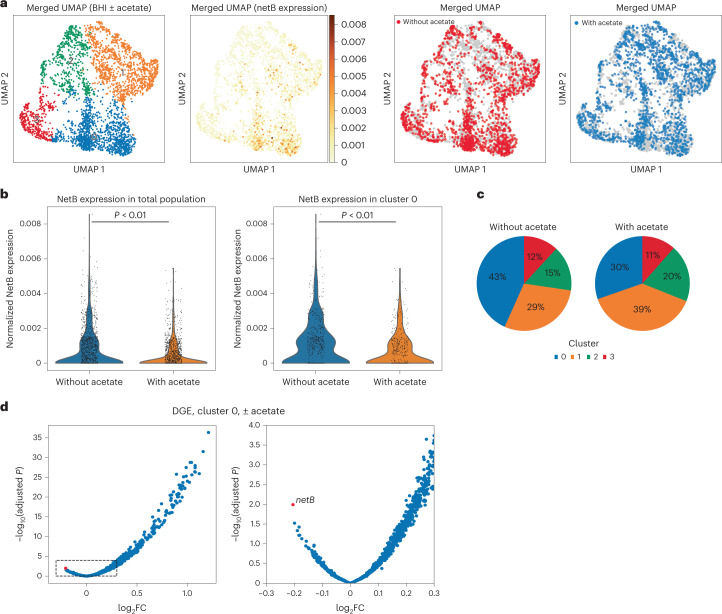
*netB* toxin in *C. perfringens* is preferentially expressed by a subpopulation of cells and can be downregulated by the addition of acetate. **a**, Merged UMAP of *C. perfringens* grown in parallel to stationary phase in BHI with and without acetate (4 mM). Far left: four populations identified after graph-based clustering. Middle left: merged UMAP of *C. perfringens* with shading representing normalized *netB* toxin expression per cell. Middle right: merged UMAP in which cells grown without acetate supplementation are highlighted in red. Far right: merged UMAP in which cells grown with acetate supplementation (4 mM) are highlighted in blue. **b**, Left: violin plot of normalized *netB* expression in all cells grown with or without acetate. *P* value < 0.01 by two-sided *t*-test. Right: violin plot of normalized *netB* expression in cells within cluster 0 grown with or without acetate. *P* value < 0.01 by two-sided *t*-test. **c**, Percentage of cells within each cluster for each condition (± acetate). **d**, Left: volcano plot of DGE of cells grown with versus without acetate supplementation. Counts normalized using DeSeq2 median of ratios method (ref. ^45^) and compared by two-sided *t*-test. Adjusted *P* values corrected by the method of Benjamini and Hochberg. Right: subset of volcano plot, focusing on genes downregulated by the addition of acetate with *netB* highlighted.

Single-cell analysis was performed on *C. perfringens* grown in rich brain heart infusion (BHI) media (Methods) to late exponential phase, when toxin is expressed. Here, we omitted inclusion of UMIs within the *C. perfringens* probe library, instead relying on the UMIs in the 10X capture oligos to quantify the number of transcripts in each cell, correcting for the overcounting induced by the in-droplet PCR (Supplementary Information and Supplementary Fig. 7). Using a custom cell-calling algorithm (Supplementary Information and Supplementary Fig. 8), 1,508 cells were resolved with an average of 507 transcripts and median of 153 transcripts detected per cell. Dimensional reduction of gene expression profiles followed by graph-based clustering revealed four distinct *C. perfringens* subpopulations (Fig. 4a). Whereas *netB* was expressed in all clusters, differential overexpression of *netB* defined cluster 0 (Fig. 4, 43% of population, log_2_FC = 1.12, *P* = 2.3 × 10^−20^, two-sided *t*-test). In fact, heterogenous netB expression was observed across three independent biological samples taken at different ODs around the time of transition from the exponential to stationary phase (Supplementary Table 6 and Supplementary Fig. 18).

### Metabolites modulate *C. perfringens* toxin expression

For *C. perfringens* in BHI, GSEA also revealed a significant enrichment of genes involved in quorum sensing (odds ratio = 6.8, *P* = 8.8 × 10^−5^, Fisher’s exact test) and fatty acid degradation (odds ratio = 33, *P* = 1.17 × 10^−5^) in cluster 0. The agr-like quorum sensing system in *C. perfringens* is required for induction of necrotic enteritis in poultry^31^ whereas production of another exotoxin, *pfoA*, has been shown to be regulated by short-chain fatty acids such as acetate and butyrate^32–36^.

Genes controlling fatty acid degradation were upregulated in the *netB*-overexpressing cluster whereas cluster 2 differentially expressed genes linked to fatty acid biosynthesis (odds ratio = 46.5, *P* = 4.3 × 10^−^^10^, Fisher’s exact test). This led us to speculate about whether toxin production and the fraction of toxin-producing cells could be regulated by specific fatty acid metabolites such as acetate. This short-chain fatty acid is abundant in the gastrointestinal tract and has been shown to inhibit *pfoA* exotoxin, which is controlled by a pH-dependent self-quorum quenching system^32–36^. Thus, we hypothesized that acetate can be used to reduce *netB* expression in *C. perfringens*.

To test this hypothesis, we perturbed the *C. perfringens* extracellular environment by adding sodium acetate to the growth medium and repeated our scRNA-seq analysis at the late exponential phase. In total, 1,575 cells were captured with an average of 676 and a median of 215 transcripts detected per cell. The two datasets (± acetate) were merged and clustered using the same parameters as applied to the original (− acetate) dataset. As before, four distinct clusters were resolved with cells from each condition dispersed throughout the clusters (Fig. 4a and Supplementary Fig. 10). Expression of *netB* was again concentrated within cluster 0 wherein cells treated with acetate expressed significantly less toxin compared to those without treatment (log_2_FC = −0.69, *P* = 1.79 × 10^−9^, two-sided *t*-test). This observation held true in the overall population as well (log_2_FC = −0.89, *P* value = 2.40 × 10^−^^23^, two-sided *t*-test). Furthermore, the addition of acetate significantly reduced the fraction of cells in the primary toxin-producing state (cluster 0) from 43% to 30% (Fig. 4c, *P* value = 9.2 × 10^−^^14^, Fisher’s exact test). This reduction in toxin gene expression was consistent with a decrease in extracellular netB protein secreted in cultures grown in media containing acetate (Supplementary Fig. 11).

Taken together, our results with *C. perfringens* demonstrate that *netB* is differentially expressed by a specialized subpopulation of cells, and that providing growth conditions favouring alternative cell states can decrease the fraction of virulent cells in a clonal bacterial population.

## Discussion

To agnostically characterize distinct transcriptional cell states within an isogenic microbial population, we developed a cost-effective bacterial scRNA-seq technique, ProBac-seq, which combines microfluidic droplet encapsulation of single cells with in situ hybridization of DNA probes for high sensitivity and throughput. Using ProBac-seq, we identified known cellular states including genetic competence and sporulation in *B. subtilis* and fimbriae production in *E. coli*. In addition, we uncovered several previously unknown transcriptional states that expressed genes that are part of metabolic pathways (amino-acid metabolism, carbon metabolism, siderophores) and physiological states (chemotaxis and motility). Strikingly, arginine biosynthesis genes appear to be preferentially expressed in distinct cell states in all three organisms that we studied.

Recently, two other methods have been proposed for scRNA-seq of bacteria: (1) sorting single bacterium into 96-well plates^7^ or (2) using a combinatorial indexing scheme on a pooled population^5,6^. Although these techniques offer advances, they also incur scalability and capture efficiency issues. For example, low numbers of detected transcripts per cell and strand-displacement activity in random hexamer primers and reverse transcription complicate accurate quantification in combinatorial indexing methods. Additionally, both methods fail to discriminate against ribosomal RNAs, comprising more than 90% of the transcriptome. Our approach requires upfront probe generation and prior knowledge of the genome, but it is fast, cheap and provides high-resolution and accurate quantification. It uses commercial equipment that is easily available and ensures sequencing is not wasted on ribosomal or other non-mRNA transcripts (Supplementary Table 8).

Several studies have reported that toxins are expressed by a subpopulation of cells among diverse bacterial species, such as *Salmonella enterica*^37^, *C. difficile*^38^, *Staphylococcus aureus*^39^ and others^40^. However, these previous studies did not identify the entire transcriptomic state of toxin-producing cells. Here ProBac-seq enabled us to identify *netB* heterogeneity and gain a transcriptome-wide view of the toxin-producing and vegetative subpopulations in *C. perfringens*. We then predicted metabolites that could alter the toxin-producing cells’ physiological state, which we confirmed by measuring toxin expression and cellular states at a single-cell resolution. We anticipate that our method will be widely used to understand how external environments modulate pathogen virulence at the single-cell level in addition to bacterial transcriptional states more broadly.

## Methods

### Strains and growth conditions

*B. subtilis* str. 168 was used for all single-cell *Bacillus* experiments. M9 was supplemented with glucose and malate together as they make up the preferred carbon source for *B. subtilis*. For experiments on suspension cultures in glucose and malate, cells were grown at 37 °C with vigorous shaking in M9 media supplemented with CaCl_2_ (0.1 mM), 0.2% glucose, tryptophan and trace metal mix^8^. Trace metal solution was made as a 100X concentrate using Na-EDTA 5.2 g, FeSO_4_-7H_2_O 2,100.0 mg, H_3_BO 330.0 mg, MnCl_2_-4H_2_O 100.0 mg, CoCl_2_-6H_2_O 190.0 mg, NiCl_2_- 6H_2_O 24.0 mg, CuCl_2_-2H_2_O 2.0 mg, ZnSO_4_-7H_2_O 144.0 mg, Na_2_MoO_4_-2H_2_O 1,200.0 mg and DI Water, 999.0 ml. The solution pH was adjusted to 7.0. At an OD of 0.4–0.5 cultures were supplemented with 50 mM malate.

*B. subtilis* strain PY79 was used for all fluorescent promoter-reporter strains. *comG* reporter strain was previously described^21^. Promoter reporters for *cotY* and *argC* were made as previously described^21^. Briefly, YFP promoter reporters were cloned into the ECE174 backbone plasmid, which uses sacA integration site and encodes chloramphenicol resistance (R. Middleton, obtained from the Bacillus Genetic Stock Center). Strains were made by genomic integration into the genome. Fluorescent reporters were integrated into the *sacA* site and checked by sequencing. The reporter strain detail is found in Supplementary Table 5. Reporter strains were grown in the same media and growth conditions used in single-cell experiments.

*E. coli* MG1655 was used in all *E. coli* single-cell experiments. For experiments in minimal media cells were grown overnight in M9 minimal media and incubated at 37 °C with moderate shaking (200 r.p.m.). Overnight cultures were diluted and subcultured and a fixed specimen was collected at mid exponential phase (OD 0.3–0.5).

*C. perfringens* strain 25037-CP01 was grown anaerobically at 37 °C in BHI media supplemented with 0.05% cysteine-HCl and, when indicated, 4 mM of sodium acetate. Anaerobic conditions were maintained using a gas-pack and anaerobic culture boxes. The oxygen indicator on all experimental replicates indicated the absence of oxygen contamination in the chamber.

### Probe set design

Probes were designed with an mRNA complementary region approximately 50 bp in length flanked by a PCR handle (18–23 bp) towards the 5′ end and a 30 poly(dA) tract on the 3′ end. On the ends of each probe, we included a region allowing for circularization and cutting with hindIII as outlined by Schmidt et al.^12^. Probe sequences are included in Supplementary Tables 1, 2 and 3.

### Probe amplification

Probes were amplified using a rolling circle method similar to the one used by Schmidt et al.^12^ with slight modifications. To amplify our probes, we did not use nicking enzymes but instead only used the HindIII digestion site in the rolling circle scheme^12^. Our incubations were scaled by a factor of five from the detailed protocol provided by Schmidt et al. Otherwise, all other aspects of the protocol were as previously detailed. Amplified probes were eluted and purified by PAGE electrophoresis using 15% urea gels and ethanol precipitation as described in Sambrook et al.^41^.

### UMI and poly-A addition

Amplified ‘proto-probes’ were extended to include a UMI and 3′ poly-adenine tail by isothermal extension with a 3′ blocked primer containing the reverse complementary sequences. In total, 100 µl of probes and 1 mg blocked extension primer (Supplementary Table 4) were mixed with 100 units of klenow fragment and 1x NEB buffer 2 and incubated for 30 min at room temperature. The extended oligonucleotide library was selectively purified by PAGE electrophoresis (final probe length approximately 142 bp) using 15% urea gels followed by ethanol precipitation as described in Sambrook et al.^42^.

### Fixation and in situ hybridization reactions and optimization

Cells from 2 ml of cell culture were fixed using a 30 min incubation in 1% paraformaldehyde (final concentration) at room temperature. Formaldehyde-fixed samples were washed with 0.02% saline sodium citrate (SSC, Invitrogen) by gentle centrifugation (6,000*g*) for 2.5 min. After the wash, cell pellets were resuspended in 1 ml MAAM (4:1 V:V dilution of methanol to glacial acetic acid). Samples were kept at −20 °C for up to 2 days before further processing. For in situ probe hybridization, 150 µl of fixed sample was centrifuged and washed once in phosphate buffered saline (PBS) to remove methanol and acetic acid. After the wash step, cells were incubated in 200 µl PBS with 350 u µl^−^^1^ of lysozyme solution (Epicentre ready-lyse) for 30 min at room temperature. Lysozyme concentration was optimized and other enzyme combinations were tested to achieve a protocol with maximal probe signal and minimal cell lysis (Supplementary Fig. 12). After 30 min, cells were pelleted and washed with 500 µl PBS-tween (PBS with 0.1% Tween 20). Cells were then resuspended with 100 µl of probe binding buffer consisting of 5 × SSC, 30% formamide, 9 mM citric acid (pH 6.0), 0.1% Tween 20, 50 ug ml^−^^1^ heparin and 10% low molecular weight dextran sulfate^42^. Cell suspensions were placed in a 50 °C shaker-incubator and allowed to pre-equilibrate for 1 hour. After 1 hour, 50 µl of probes (600 ng µl^−1^) were added to each cell suspension and samples were left to incubate overnight. After the overnight incubation samples were washed five times in prewarmed (50 °C) probe-wash solution (5 × SSC, 30% formamide, 9 mM citric acid pH 6.0, 0.1% Tween 20 and 50 ug ml^-1^ heparin). Before encapsulation on the 10X device, cells were washed three times in PBS and diluted as suggested in the 10X Chromium instruction manual. A flow cytometer was used to evaluate the retention of cells through a typical proBac-seq probe hybridization and wash procedure. Approximately 90% of fixed cells remained in solution at the end of the protocol (Supplementary Fig. 13).

### Microfluidic encapsulation and droplet generation reaction

Single-cell partitioning, barcoding and cDNA library generation was achieved using the 10X Genomics Chromium Controller with the Chromium Single Cell 3′ Reagents Kit (v2 chemistry) as described by 10X Genomics (https://support.10xgenomics.com/permalink/user-guide-chromium-single-cell-3-reagent-kits-user-guide-v2-chemistry). The protocol was modified to achieve bacterial scRNA-seq. For GEM generation (10X microfluidic encapsulation), a master mix containing the following reagents (per rxn, not accounting for excess volume) was prepared: 33 µl of 4X ddPCR Multiplex Supermix (BioRad), 4 µl of custom primer (10 µM), 2.4 µl additive A (10X Genomics) and 26.8 µl dH_2_O. All other reagents specified by 10X Genomics were omitted. Prepared cell samples were washed three times in PBS and diluted to 1,000 cells µl^−^^1^ before loading on the microfluidic chip (‘Chip A Single Cell’) with a targeted cell recovery of 10,000 cells. Our recovery was lower than the expected cell number and we attribute this to the difficulty in obtaining accurate cell number measurements when working with small numbers of fixed bacterial cells.

### Library construction and sequencing

After microfluidic encapsulation on the Chromium Controller, each sample (that is ‘reaction’) was visually inspected to confirm successful GEM formation (should observe a well distributed emulsion with a volume of approximately 100 µl). Samples were then transferred to fresh PCR tubes and cycled at the following conditions (replacing the ‘GEM-RT Incubation Step’ in 10X Genomics protocol): 94 °C for 5 min, six cycles of 94 °C for 30 s followed by 50 °C for 30 s then 65 °C for 30 s, held at 4 °C.

After PCR one, the emulsion was broken and the pooled DNA purified using Dynabeads MyOne Silane as described in 10X Genomics’ protocol. Purified DNA was amplified once more (replacing the ‘cDNA Amplification step’ of 10X Genomics’ protocol) using a master mix composed of 17 µl of purified DNA, 20 µl of Q5 Hot Start 2X MM, 1.5 µl of forward primer (10 µM) and 1.5 µl of reverse primer (10 µM). PCR conditions included a 30 s incubation at 98 °C followed by 16 cycles of 10 s at 98 °C, 20 s at 62 °C and 20 s at 72 °C before a final extension at 72 °C for 2 min. After PCR two, amplified DNA was purified using the NucleoSpin Gel and PCR Clean-up kit (Macherey-Nagel) as per manufacturers’ instructions. Purified DNA was run on Agilent TapeStation to confirm the presence of a band at 180 bp, indicating successful library generation. Libraries were then prepared for sequencing by Illumina adaptor addition via low cycle (*n* = 6) PCR with custom library preparation finishing primers (Supplementary Table 3).

Sequencing was carried out on an Illumina nextSeq-1000 instrument with 100 cycle reagents. Libraries were spiked with 30% PhiX and sequenced for 8 bp in the i7 index direction and 119 bp for ‘Read 1’. Addition of 30% PhiX improved fastQ quality scores.

### RNA-seq of bulk samples

*B. subtilis* RNA-seq libraries used for comparison of traditional RNA-seq versus probe-based transcriptomics were first fixed with 1% paraformaldehyde for 30 min in room temperature. After fixation cells were pelleted by centrifugation (6,000*g*) for 5 min and formaldehyde was removed. Pellets were washed in PBS buffer. Washed cells were rehydrated in 240 µl Qiagen PKD buffer (FFPE miRNEASY kit, Qiagen). Cells were lysed by the addition of 10 µl lysosome solution for 10 min followed by bead beating using the Bullet Blender Gold and RINO beads as recommended for *B. subtilis* samples by the manufacturer. Lysed samples were further processed using the Qiagen FFPE miRNA kit. Libraries were prepared from total RNA (without removal of ncRNA) using the Takara Smart-Seq Stranded kit as per the manufacturer’s instructions.

*E. coli* RNA-seq libraries used for comparison of traditional RNA-seq versus probe-based transcriptomics were first stabilized in Qiagen RNAprotect Bacteria reagent (or fixed in paraformaldehyde using standard single-cell protocol for half the sample that underwent probe-based analysis). RNA extraction from stabilized pellets was carried out using the standard Qiagen RNeasy kit and the recommended protocol for *E. coli* provided in the Qiagen RNAprotect Bacteria Reagent Handbook (v.HB-1704-002). Libraries were prepared from total RNA (without removal of ncRNA) using the Takara Smart-Seq Stranded kit as per the manufacturer’s instructions.

Raw, demultiplexed reads were trimmed to remove adaptor regions and aligned to the relevant reference transcriptome or probe library using Bowtie2. Alignments were enumerated with featureCounts using the appropriate strandedness argument depending on the method of library preparation (Smart-seq stranded kit (Takara) or proBac-seq (this article)). Count matrices (in the form of reads per gene or probe, or reads per gene or probe per cell) were then used to quantify gene expression. For RNA libraries prepared by random priming, reads were normalized by transcript length and sequencing depth as reads per kilobase per million reads.

### Microscopy

Cultures were visualized on a Leica DM3000 light microscope before single-cell experiments to ensure samples were free of chains and clumps. Reporter strains were imaged using a ×100 oil-immersion objective on a Zeiss Axio Observer inverted microscope equipped with a colibri-7 light emitting diode (LED) fluorescent light source and an axiocam digital camera. Flagellar staining was done using the Remel flagellar stain (Thermo Fisher Scientific) as per manufacturer instructions.

### Image analysis using cell segmentation and outlier identification

Cells in microscopy images were segmented using the microbeJ program within FIJI (ImageJ). Outliers were identified by using the IQR outlier method. Specifically, quartiles and the IQR were determined for each dataset. Outliers were determined by standard outlier detection parameters: cells that were more than 1.5 IQRs above quartile 3 were designated as cells within an overexpressing population. Population histograms were plotted and outliers reported.

### Western blot analysis

To collect conditioned media from *C. perfringens* cultures at OD_600_ corresponding to late exponential growth (OD_600_ between 0.7 and 0.8) were pelleted by centrifugation at 4,200*g* for 4 min. Supernatant was then filtered through a 0.2 µM filter. Then 5 µl filtered conditioned media was incubated with NuPage LDS loading buffer (Invitrogen) for 10 min at 95 °C. Samples were loaded in equal volume and run on a 4–12% Bis Tris polyacrylamide gel. PAGE gels were transferred to an invitrolon 45 µM polyvinylidene difluoride membrane that was presoaked in methanol using the Xcell-II blot apparatus (Invitrogen) as per the manufacturer’s instructions. Toxin netB (33 Kd) was detected using custom polyclonal rabbit antibodies (ProSci, Poway CA) at a 1:1,000 dilution and the WesternBreeze rabbit chromogenic Western Blot kit (Invitrogen). We used the SeeBlue Plus2 prestained ladder (Novex, Life Technologies) as a marker of protein size. All experiments were done using at least biological triplicates and technical triplicates on numerous independent days and representative images were selected.

### Transcriptomic analysis and visualization

Single-cell gene expression matrices, *c*^*g*^, were analysed with Seurat (v.3.1.2 with default parameters except where indicated). We first log-transformed the data using the ′NormalizeData(normalization.method = ‘LogNormalize’, scale.factor = 10000)′ function, and selected the 2000 most variable genes using ′FindVariableFeatures(selection.method = ‘vst’, nfeatures = 2000)′. Then, we *z*-scored these highly-variable genes using ′ScaleData′. Next, we performed linear dimensionality reduction using principal component analysis (PCA) down to 50 dimensions (′RunPCA′). Points in this embedding were used to construct UMAP plots (′RunUMAP(dims = 1:10)′) and find neighbours for clustering (′FindNeighbors′). ′FindClusters(resolution = 1.0)′ was used to run the Louvain clustering algorithm and generate clusters. We confirmed that the clusters highlighted in the main text appeared consistently for a range of resolutions from 0.5 to 1.5. Summary statistics of the number of genes/transcripts in a cell and cluster populations for the different samples can be found in Supplementary Table 8. DGE was performed using the ′FindMarkers(min.pct = 0.25)′ function with a log-fold change cutoff of 0.25, whose output when applied to our data is shown in the DGE Supplementary Tables. Heatmaps of differentially expressed genes were generated using the ′DoHeatMap′ function using the standard Seurat pipeline, which displays the genes with highest scores as markers of each given cluster and show only the top overexpressed genes in each cluster with Bonferroni-corrected *P* value < 0.05. Other differentially expressed genes found in each cluster can be found in the supplementary DGE tables. For *C. perfringens* data, the above computational pipeline was repeated analogously using the Scanpy package^43^ except for the following three steps: PCA was performed using all genes; DGE was performed with ‘rank_genes_groups’ function which uses two-sided *t*-test; the PAGA algorithm was run with the default parameter to obtain a graph for seeding UMAP; and we ran UMAP with the minimum distance and spread parameters set to 0.3 and 5, respectively.

### Calculating corrected multiplet rate

A ‘barnyard experiment’ was conducted in which *E. coli* and *B. subtilis* cells were mixed at a target species ratio of 1:1 and analysed by our bacterial single-cell RNA-seq method using the 10X Chromium Controller. We recovered 3,373 GEM containing droplets with 45 (1.3%) showing evidence of interspecies collisions based on reads sharing the same cellular barcode but mapping back to different species probe sets. Assuming an exact 50:50 species mix, the true multiplet rate (accounting for both inter- and intraspecies collisions) is then estimated as 2 × 1.3%, or 2.6%; that is, in experiments containing a single bacterial species, 2.6% of called ‘cells’ would actually represent cell multiplets. We can further correct this estimate based on the observed species ratio, following Bloom^44^. In the same dataset, the number of droplets containing at least one *E. coli* cell (N1) was found to be 2,095 based on mapped reads and the number of droplets containing at least one *B. subtilis* cell (N2) was 1,323 for an observed species ratio of ~1.6:1. The number of droplets containing at least one cell of each species (N1,2) is 45, as before. Therefore, the true corrected multiplet rate can be analytically determined as 2.8%. For comparison, 10X Genomics reports an expected multiplet rate of 2.4% for 3,000 recovered cells based on the anticipated number of droplets generated per lane and Poisson loading.

### Reporting summary

Further information on research design is available in the Nature Portfolio Reporting Summary linked to this article.

### Supplementary information

Supplementary Information Supplementary Information.

Reporting Summary

Supplementary Tables 1–14 Supplementary Table 1: *B. subtilis* probes ordered from TWIST Bioscience. Supplementary Table 2: *E. coli* probes ordered from TWIST Bioscience. Supplementary Table 3: *C. perfringens* probes ordered from TWIST Bioscience. Supplementary Table 4: Probe amplification reagents and costs. Supplementary Table 5: Strains used in this study. Supplementary Table 6: DGE analysis. Supplementary Table 7: Gene clusters list. Supplementary Table 8: Sample summary. Supplementary Table 9: DGE analysis using 10X UMIs instead of probe UMIs. Supplementary Table 10: DGE analysis using bulk median instead of per cell maximum probe counts. Supplementary Table 11: Genes in each section of the Venn diagram in Fig. 3d. Supplementary Table 12: Genes used in volcano plots on Fig. 2c and Supplementary Figs. 3 and 6. Supplementary Table 13: Gene annotations for locus tags in Supplementary Figs. 9 and 10. Supplementary Table 14: Sequencing depth and percent reads mapping to probes for each sample.

## Data Availability

All sequence data used in this publication are publicly available through the National Center for Biotechnology Information’s GEO repository under accession number GSE223752. The genome for *Clostridium perfringens* has been deposited in the National Center for Biotechnology Information under the accession numbers CP109957–CP109962.

## Code availability

Code to prepare custom references, reformat FASTQ files for the CellRanger pipeline, generate single-cell gene expression matrices from single-cell probe expression matrices, correct for spurious UMIs in *C. perfringens* data and perform custom cell-calling is available at https://gitlab.com/hormozlab/bacteria_scrnaseq.
