# Supplementary material for: Probe-based bacterial single-cell RNA sequencing predicts toxin regulation
Source: Nat Microbiol. 2023 Apr 3;8(5):934–45. doi: 10.1038/s41564-023-01348-4 (PMC10159851; doi:10.1038/s41564-023-01348-4)
Supplement: Supplementary file 1 — Supplementary Information. [file 41564_2023_1348_MOESM1_ESM.pdf]

nature microbiology

Article

https://doi.org/10.1038/s41564-023-01348-4

Probe-based bacterial single-cell RNA sequencing predicts toxin regulation

In the format provided by the authors and unedited

Supplementary Figures

Figure S1: Genome wide scRNA-seq probe design and synthesis

Figure S2: Detailed schematic of C2C-based proto-probe amplification and primer extension incorporation of probe-based UMI and polyA

Figure S3: Heatmap of *B. subtilis* cells from Figure 2 including all marker gene names

Figure S4: Frequency of marker genes and spores in the population measured by reporter strains

Figure S5: ProBac-seq analysis of gene distance matrices is able to identify genes with architectural similarity, including genes within operons and regulons

Figure S6: Heatmap of aerobic M9 culture of *E. coli* cells from Figure 3 including all marker gene names

Figure S7: Estimating normalization factor for removing spurious UMI

Figure S8: Cell-calling algorithm for *C. perfringens* datasets generated with ProBac-seq

Figure S9: Heatmap of *C. perfringens* grown in BHI media with no added acetate from Figure 4 including all marker gene names or genome locus tags – to accompany data in Figure 4

Figure S10: Heatmap of *C. perfringens* grown in BHI media with 4mM acetate from Figure 4 including all marker gene names or genome locus tags – to accompany data in Figure 4

Figure S11: Presence of acetate in the culture media reduces the level of extracellular NetB toxin

Figure S12: *In situ* Hybridization protocol optimization reveals that addition of the cell wall degrading enzyme lysozyme helps increase probe signal

Figure S13: Over 90% of cells are retained after ProBac-seq in-situ probe hybridization and washes

Figure S14: scRNAseq of *B. subtilis* using 10X UMIs instead of probe UMIs

Figure S15: scRNAseq of *E. coli* in aerobic M9 culture using 10X UMIs instead of probe UMIs

Figure S16: scRNAseq of *B. subtilis* using bulk median instead of per-cell maximum probe counts for gene expression

Figure S17: scRNAseq of *E. coli* cells in aerobic M9 media using bulk median instead of per-cell maximum probe counts for gene expression

Figure S18: Reproducibility of biological replicates grown in BHI condition

Figure S19: Probe based transcriptomic measurement correlates strongly with traditional RNAseq transcriptomic measurement

Figure S20: scRNAseq of *B. subtilis* using in-droplet reverse transcription (RT) instead of in-droplet PCR identifies sporulation and competence populations

Figure S21: *In situ* Hybridization optimization for *Clostridium perfringens*

Supplementary Protocols

Supplementary Protocol 1: Constructing a Reference Genome

Supplementary Protocol 2: Creating FASTQ Files for CellRanger

Supplementary Protocol 3: Comparing Single cell Probe Expression Matrices Generated Using 10X vs Probe UMIs

Supplementary Protocol 4: Single cell Gene Expression Matrices from Single cell Probe Expression Matrices

Supplementary Protocol 5: Removal of spurious UMI

Supplementary Protocol 6: Cell calling

Supplementary Notes

Supplemental note 1: Additional biology related to Figure 2 and Figure S3

Supplemental note 2: Benchmarking ProBac-Seq probe-based transcriptomics to traditional RNAseq

Supplemental note 3: Additional biology related to Figure 3 and Figure S6

Supplemental note 4: Additional info on Figure S20 and use of droplet RT instead of PCR

Supplemental note 5: Calculation of probe concentrations

Supplemental note 6: Probe design approach and limitations, probe analysis and ProBac seq protocol optimization

Supplemental note 7: Reproducibility of biological replicates

Supplemental note 8: Use of 10X microfluidic encapsulation with small particles

Figure S1: Genome wide scRNA-seq probe design and synthesis

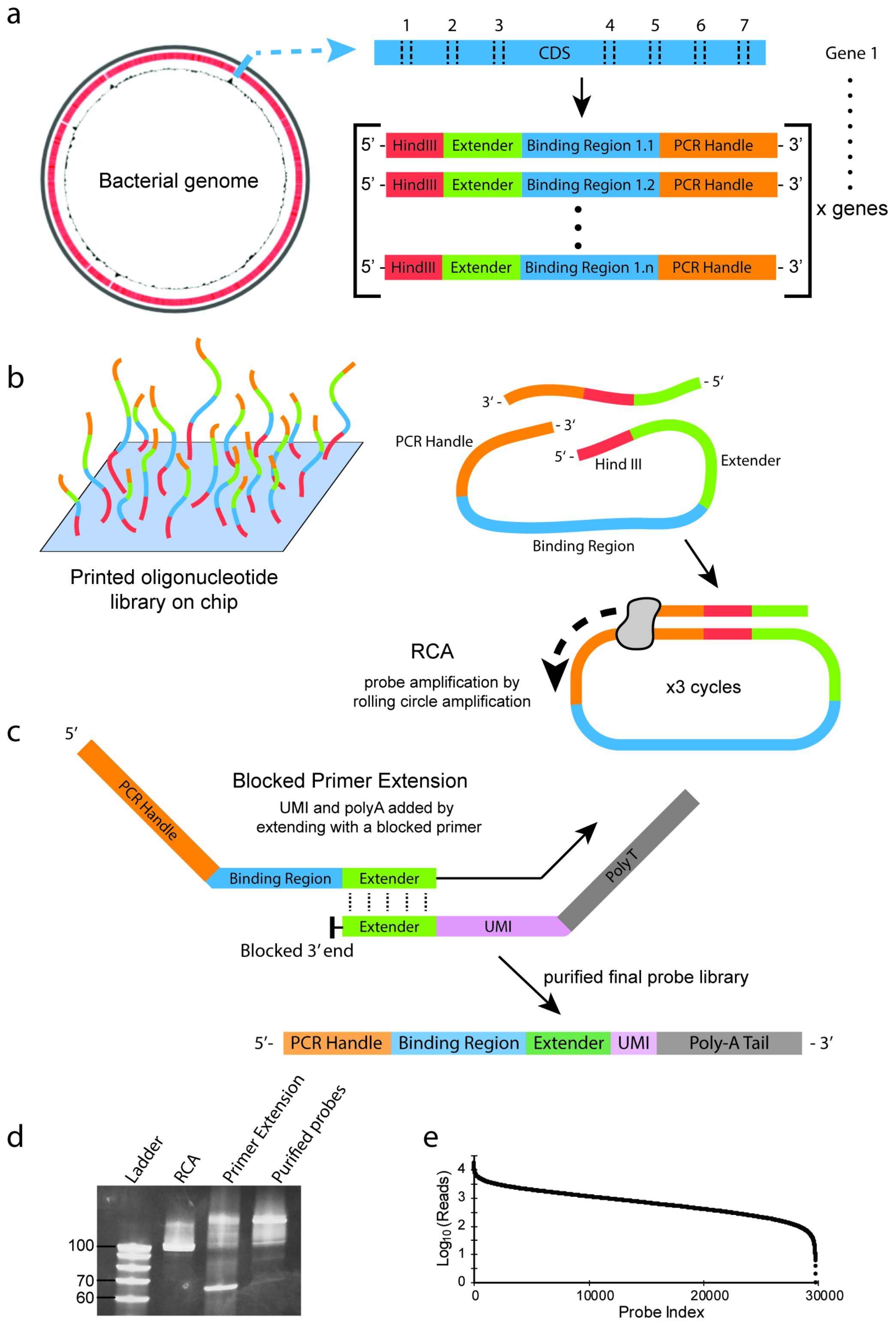

a. A genome for the organism of interest is used to design a library of probes that are complementary to unique regions within each gene's coding sequence (CDS). Complementary ssDNA oligonucleotides are amended by addition of an extender sequence at the 5' end and 3' PCR handle. These sequences are ordered as an oligo pool from TWIST Biosciences **b.** Probe sequences are inverted in-silico and restriction sites and handles are added for circle-to-circle amplification. The large, inverse oligonucleotide library is commercially synthesized and pooled. Ordered oligo-pools are amplified in a 3-step "rolling circle" reaction with Phi29 polymerase to produce the final, correctly oriented proto-probe library. **c.** A random 12mer sequence (UMI) and 30-base polyA tail is added by primer extension with a blocked primer that is complementary to the 3' extender sequence. Single stranded DNA (ssDNA) containing a universal PCR handle, UMI, complementary transcript region, and poly-A tail (listed 5' to 3') is purified. **d.** Denaturing ssDNA gel shows length of RCA product and final probes after addition of 3' end. Libraries run along alongside a ssDNA ladder from IDT (60-100 bp markers, leftmost lane). Finalized probe libraries are free of amplification primers **e.** Distribution of probes in final library – data shown for *B. subtilis* probe library.

Figure S2: Detailed schematic of C2C-based proto-probe amplification and primer extension incorporation of probe-based UMI and polyA

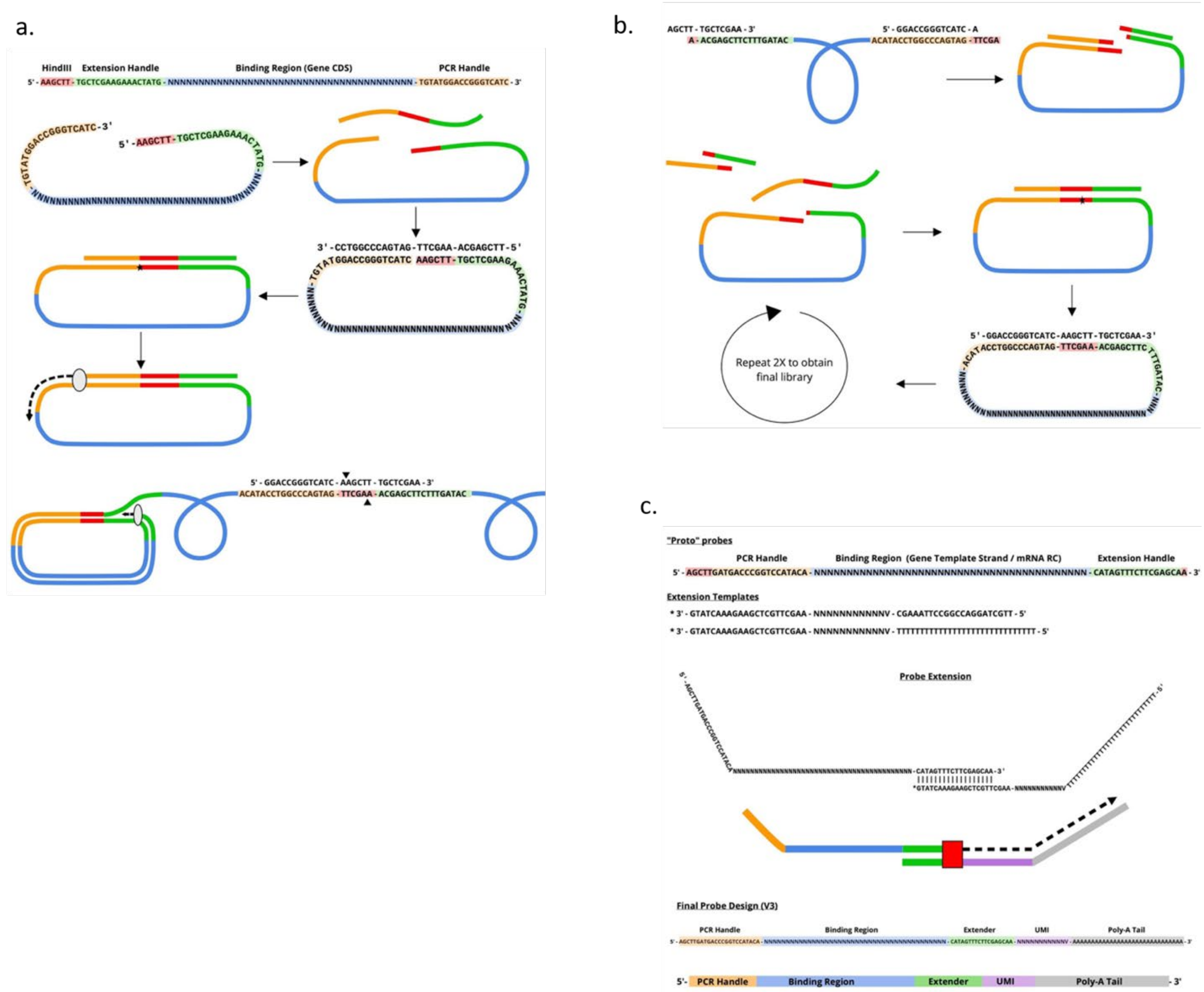

a. Oligonucleotide pools ordered from TWIST biotech were circularized by addition of a scaffolding primer that contained a sequence with reverse-complementarity to the two adjoining ends. Circles were finalized with T4 ligase and the scaffolding primer was used as a primer for phi29-mediated rolling circle amplification. **b.** Amplified circles were cut into single-strand primers by addition of nicking primer with reverse complementarity to the hindIII containing region and the two end-joints. Circles were made with these newly liberated single-strand amplified probes by adding the appropriate reverse-complement primer and ligation and the phi29 reaction was carried out as before. Libraries were amplified in 3 total rounds of rolling circle reactions. **c.** After digestion of the products from the third and final rolling circle amplification proto-probes were purified by gel electrophoresis and a UMI and polyA tail were added by primer extension using a blocked extension template.

Figure S3: Heatmap of *B. subtilis* cells from figure 2 including all marker gene names

a

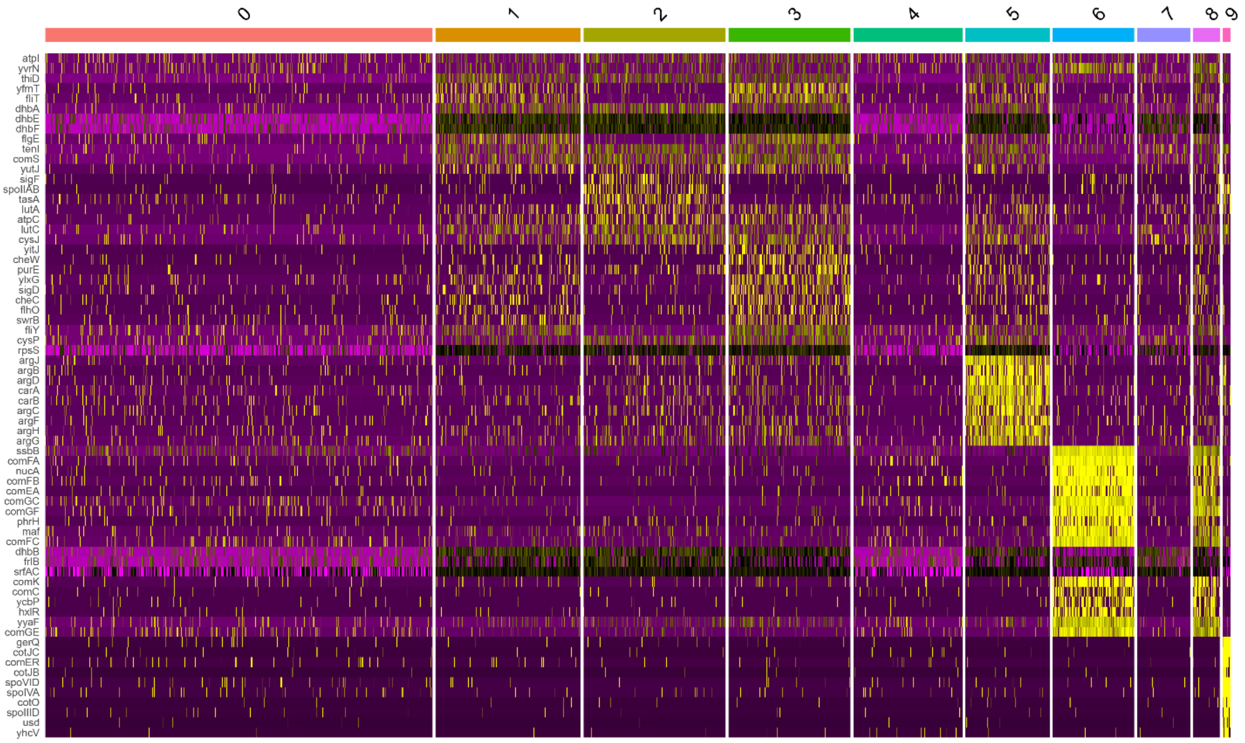

b

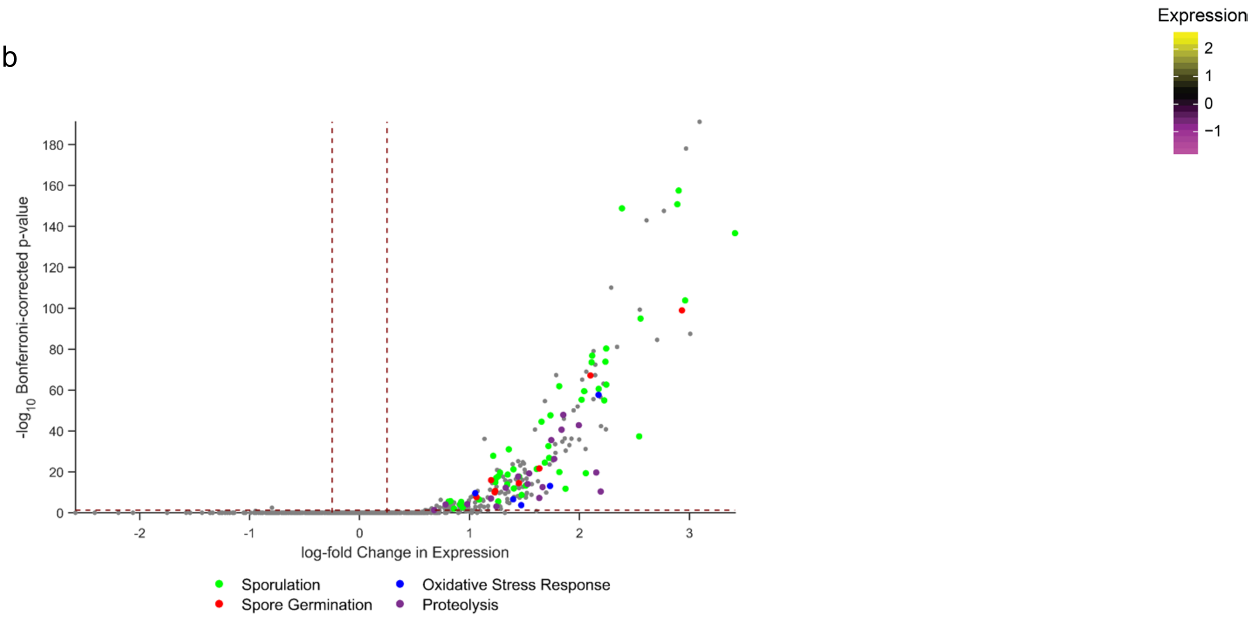

a. Heatmap as in figure 3a. including names for all marker genes
b. Gene enrichments in cluster 9 represent sporulation genes as in Figure 2c as well as other gene-sets enriched in this cluster and highlighted in this panel. Volcano plot generated from results of DGE analysis in Seurat (two-sided Wilcoxon rank sum test with Bonferroni correction)

Figure S4: Frequency of marker genes and spores in the population measured by reporter strains

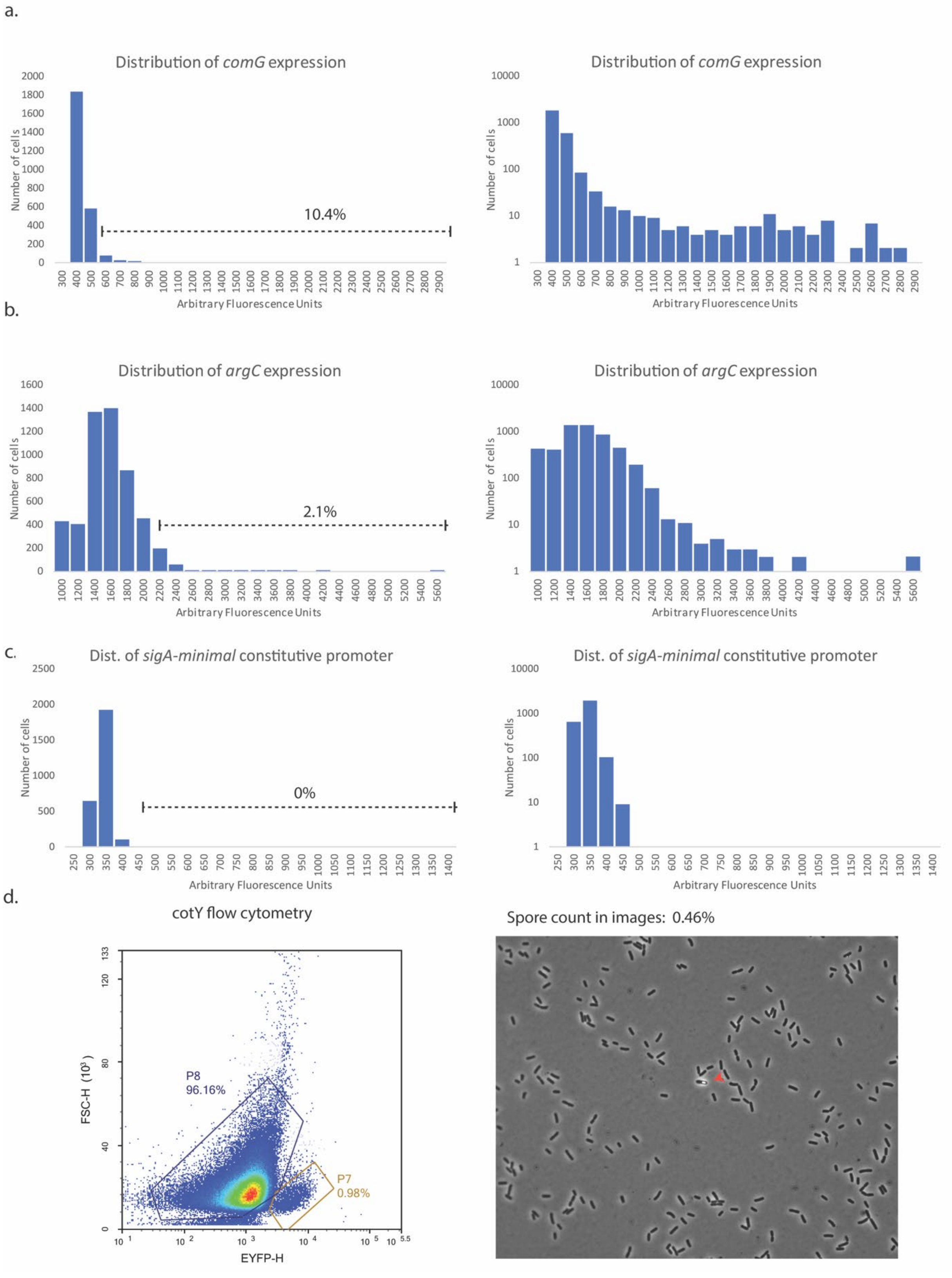

a. Approximately 10.4% of the population is in the high-expressing *comG* tail of the distribution (*comG* promoter-reporter strain fluorescence per cell – determined by 1.5x IQR – see Methods). In order to show small cell numbers in the tail more clearly, the histogram is graphed using both a standard scale (left) and a $\log_{10}$ scale (right) for the number of cells in each bin. This is the same strain used in previous studies (Rosenthal et al, eLife) with similar distributions **b.** Approximately 2.1% of the population is in the high-expressing *argC* tail of the distribution (1.5x IQR). In order to show small cell-numbers in the tail more clearly, the histogram is graphed using both a standard scale and a $\log_{10}$ scale **c.** A minimal constitutive promoter reporter (sigA) does not have a long-tailed distribution. To show the lack of a long tail the X axis was extended **d.** Sporulation marker gene *cotY* is expressed in approximately 1% of cells (P7 depicts fluorescence from this promoter-reporter strain in the flow cytometry panel, left) and spores are present in a small fraction of cells (approx. 0.5% - right panel)

Figure S5: ProBac-seq analysis of gene distance matrices identifies covarying genes, including genes within operons and regulons

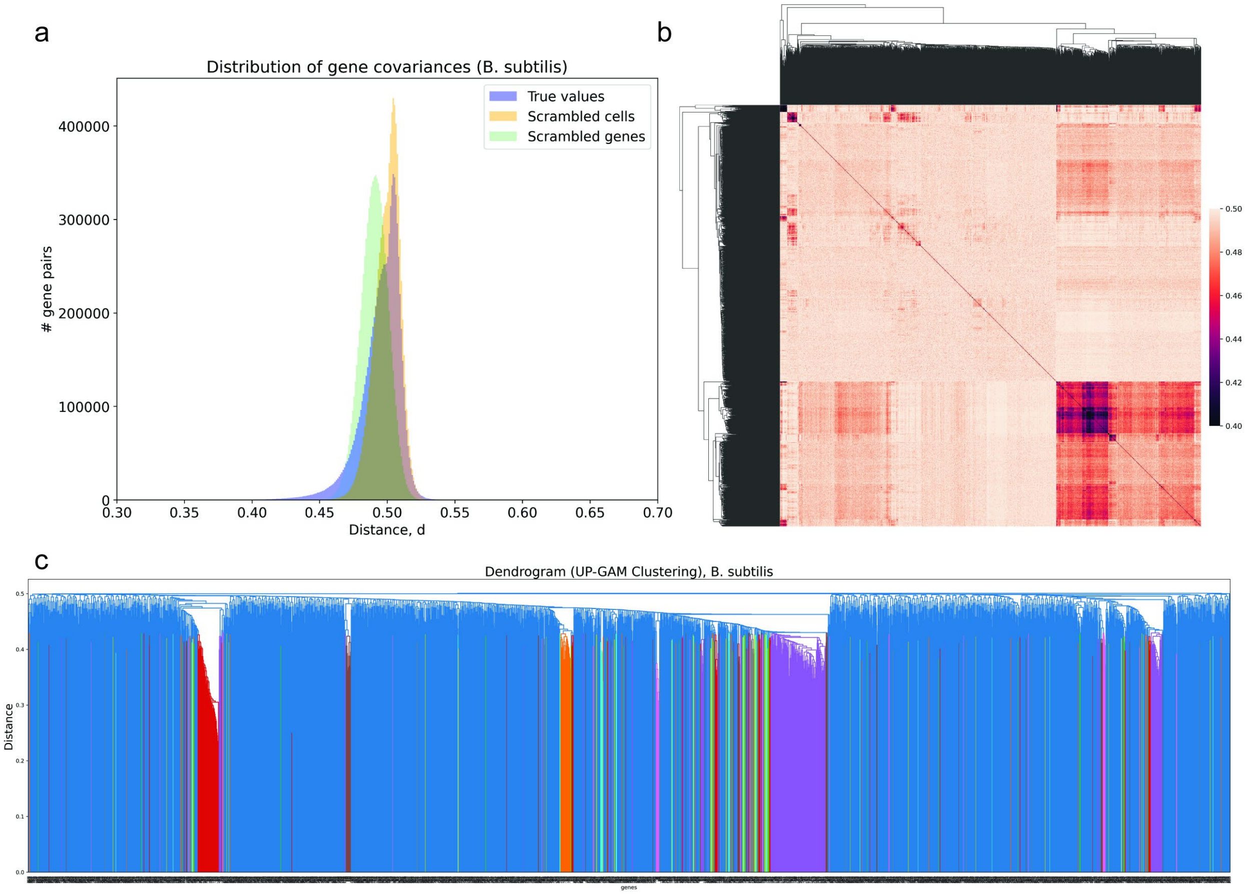

a. Distribution of spearman distances from all gene pairs calculated from ProBac-Seq single-cell dataset of *B. subtilis* in minimal media. **b.** Clustered heatmap of gene distances. **c.** Dendrogram of gene distances after hierarchal clustering using average linkage (UP-GAM). Gene pairings with distance value < 0.43 (empirically chosen) are highlighted. Lists of genes clustering together can be found in Supplementary Table S7

Figure S6: Heatmap of aerobic M9 culture of *E coli* cells from figure 3 including all marker gene names

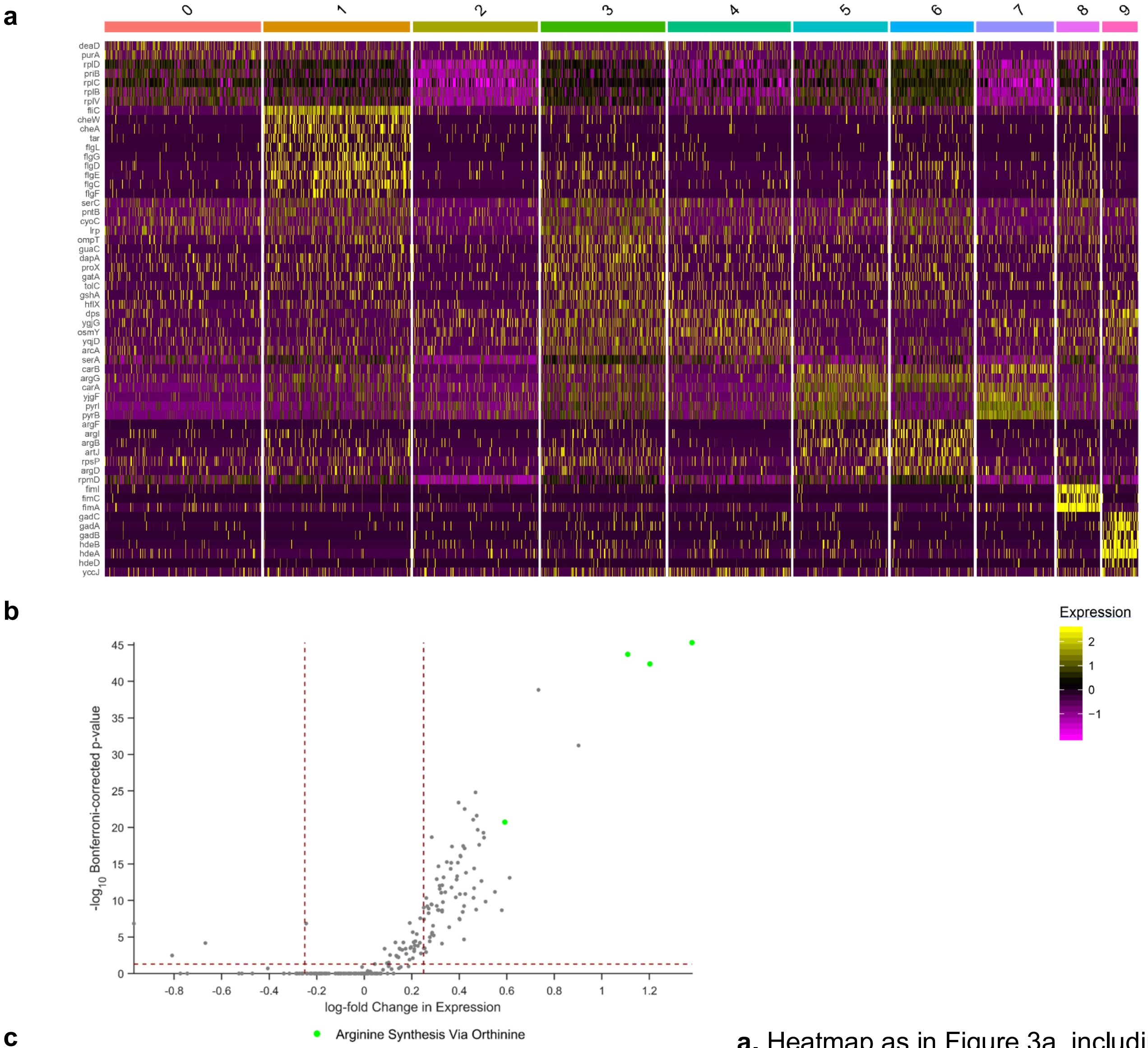

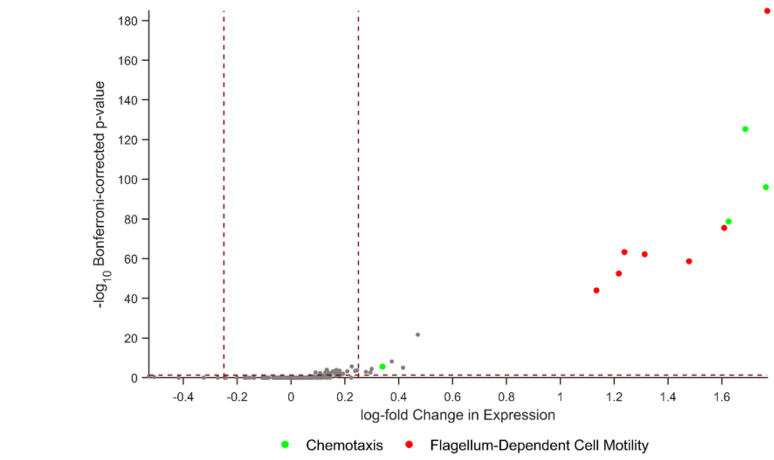

a. Heatmap as in Figure 3a. including names for all marker genes. Values are z-scores of log-transformed values
b. Volcano plot showing gene expression in cluster 6 is enriched for arginine synthesis genes. P-values from two-sided Wilcoxon rank sum test with Bonferroni correction.
c. Volcano plot showing gene expression in cluster 1 is enriched for flagella and chemotaxis. P-values from two-sided Wilcoxon rank sum test with Bonferroni correction.

Figure S7: Estimating normalization factor for removing spurious UMI

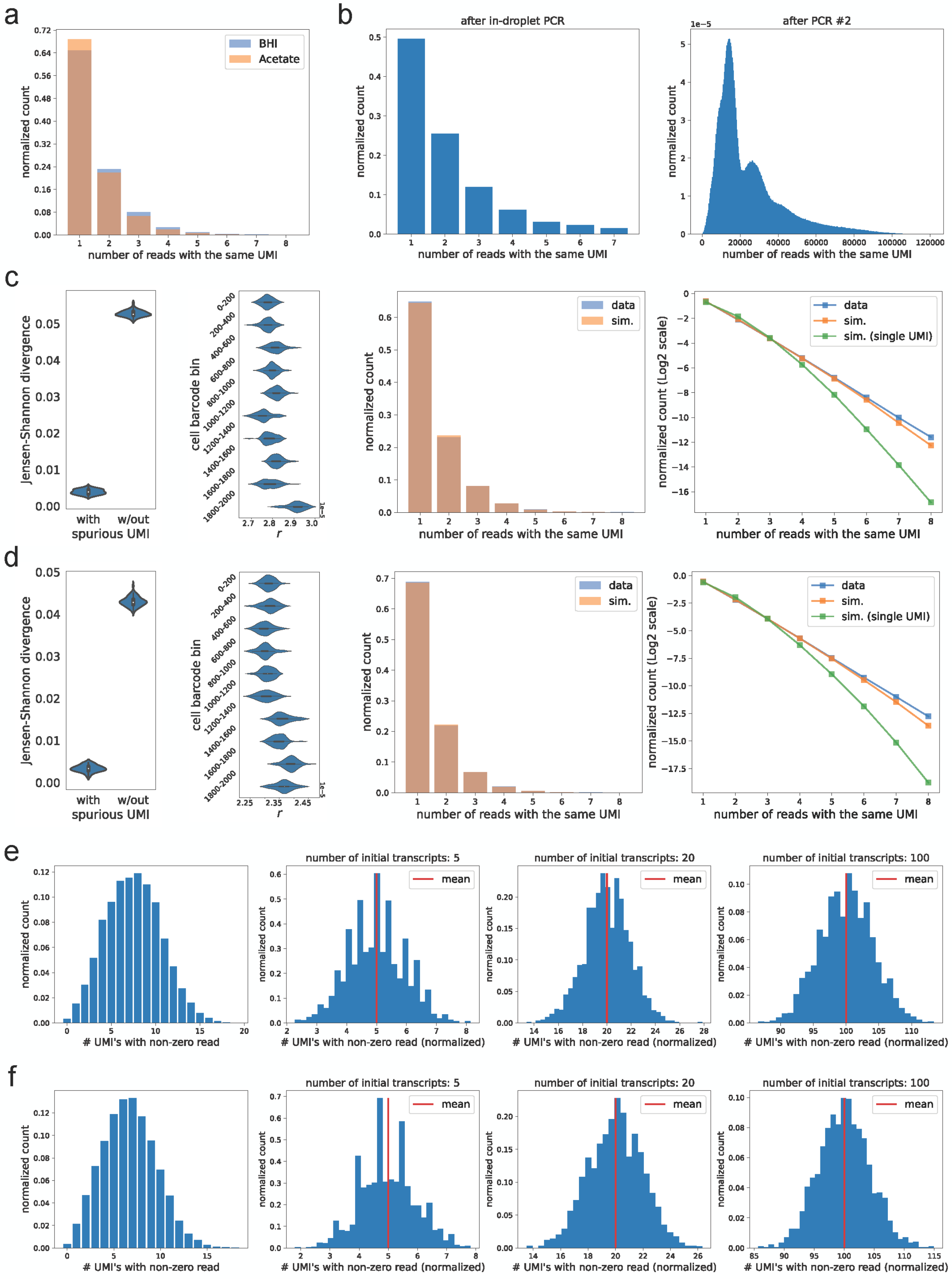

a. Normalized histogram of UMI count, truncated at 8 reads and aggregated over top 2000 cell barcodes with the largest number of total reads in the BAM file. The histograms in both BHI and BHI + acetate conditions exhibit rapid decay. **b.** Normalized histogram of simulated UMI count after in-droplet PCR (left) and after out-of-droplet PCR (right). The bar heights of in-droplet PCR decrease roughly by half because 10X primers, which introduce new UMIs, can land only on reverse strands at each cycle. Each bar gets amplified and spreads out, due to the variance of binomial draws, during 16 cycles of out-of-droplet PCR. For example, the peak of the right histogram, at ~15000 reads, comes from the superposition of reads amplified from the first two bars in the left histogram. **c.** Optimization of subsampling factor for BHI condition data showing, from left to right, violin plots of Jensen-Shannon divergence at a converged value of subsampling factor r using simulation with and without spurious UMIs (1st), violin plots of the optimal value of r at each of 200 cell barcode bins (2nd), overlay of the normalized data histogram from a and normalized histogram of aggregated UMI counts subsampled with r=2.77e-5 (3rd), the same histograms shown in log2 scale, with the addition of normalized histogram from simulation where in-droplet PCR produces a single UMI with 64 reads. The simulation without spurious UMIs does not fit the data well because subsampled UMI counts decay much faster. The optimal values of r are stable across bins. This result indicates that the degree of subsampling is the same across all cells regardless of the total number of UMIs observed in the cells. **d.** Optimization of subsampling factor for BHI + acetate condition data. The mean of the optimal values of r is 2.33e-5. The optimal value of r increases for bins with cell barcodes that have a smaller number of total reads. This trend is consistent with the cell-calling result where 1575 out of the top 2000 cell barcodes were called as valid cells. **e.** Estimation of normalization factor for BHI condition data showing, from left to right, normalized histogram of the number of UMIs that resulted from a single transcript with non-zero UMI count after subsampling, aggregated over 50 100000 trials (1st), normalized histogram of the number of UMIs with non-zero UMI count after subsampling, aggregated over 1000 trials starting from small, intermediate, and large numbers of initial transcripts, divided by the mean of the first histogram (next 3 panels). Subsampling is performed with r=2.77e-5, and the normalization factor is 7.49. **f.** Estimation of normalization factor for BHI + acetate condition data. Subsampling is performed with r=2.33e-5, and the normalization factor is 6.65. In both conditions, the mean of normalized numbers of UMI recovers the true number of initial transcripts for all three expression levels.

Figure S8: Cell-calling algorithm for *C. perfringens* datasets generated with ProBac-seq

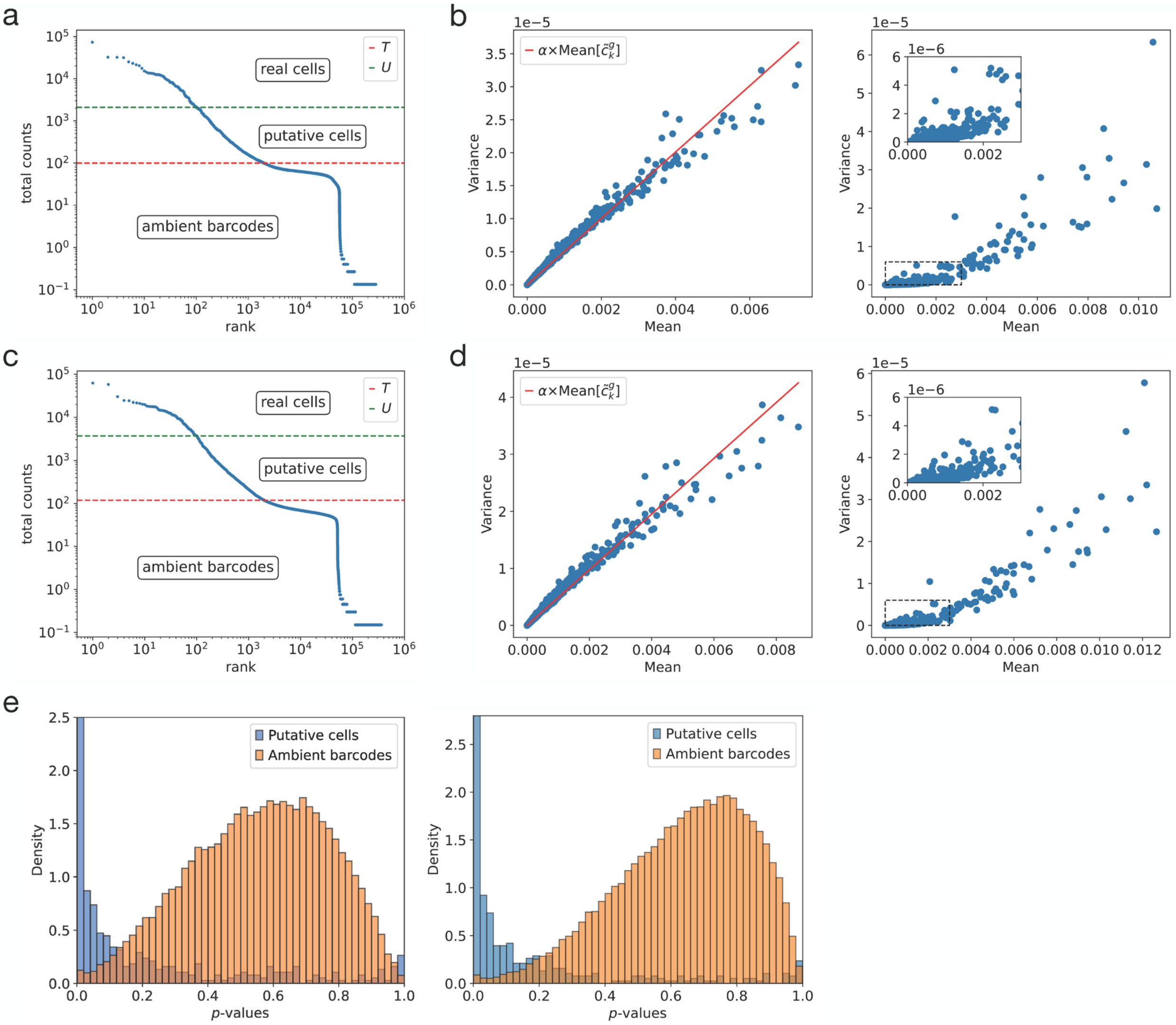

a. Barcode rank plot of normalized count matrix for BHI condition data. We divided cell barcodes into three types: real cells, putative cells, and ambient barcodes, using an upper threshold U and a lower threshold T. The first 100 cell barcodes were selected as real cells and the next 1900 cell barcodes as putative cells. **b.** Variance v.s. mean of expression frequencies of ambient barcodes (left) and real cells with lowly expressed genes magnified in the inset (right) for BHI condition data. Variance and mean show a clear linear trend in ambient barcodes whereas variance grows faster than linearly in real cells. The fitted value of proportionality constant is 5.02e-3. **c.** Same as **a.** for BHI + acetate condition data. **d.** Same as **b.** for BHI + acetate condition data. The fitted value of is 4.88e-3. **e.** Normalized histogram of p-values from putative cells and ambient barcodes with respect to the negative binomial null model for BHI condition data (left) and BHI + acetate condition data (right). Y-axes of both figures are magnified to highlight the spread of the p-values of ambient barcodes. The density values of the first bar, p-value of 11001, are actually 42.5 and 43.5 for BHI and BHI + acetate data, respectively. All real cells yielded the lowest p-value in both data and are thus not shown.

Figure S9: Heatmap of *C. perfringens* grown in BHI media with no added acetate from figure 4 including all marker gene names or genome locus tags – to accompany data in figure 4

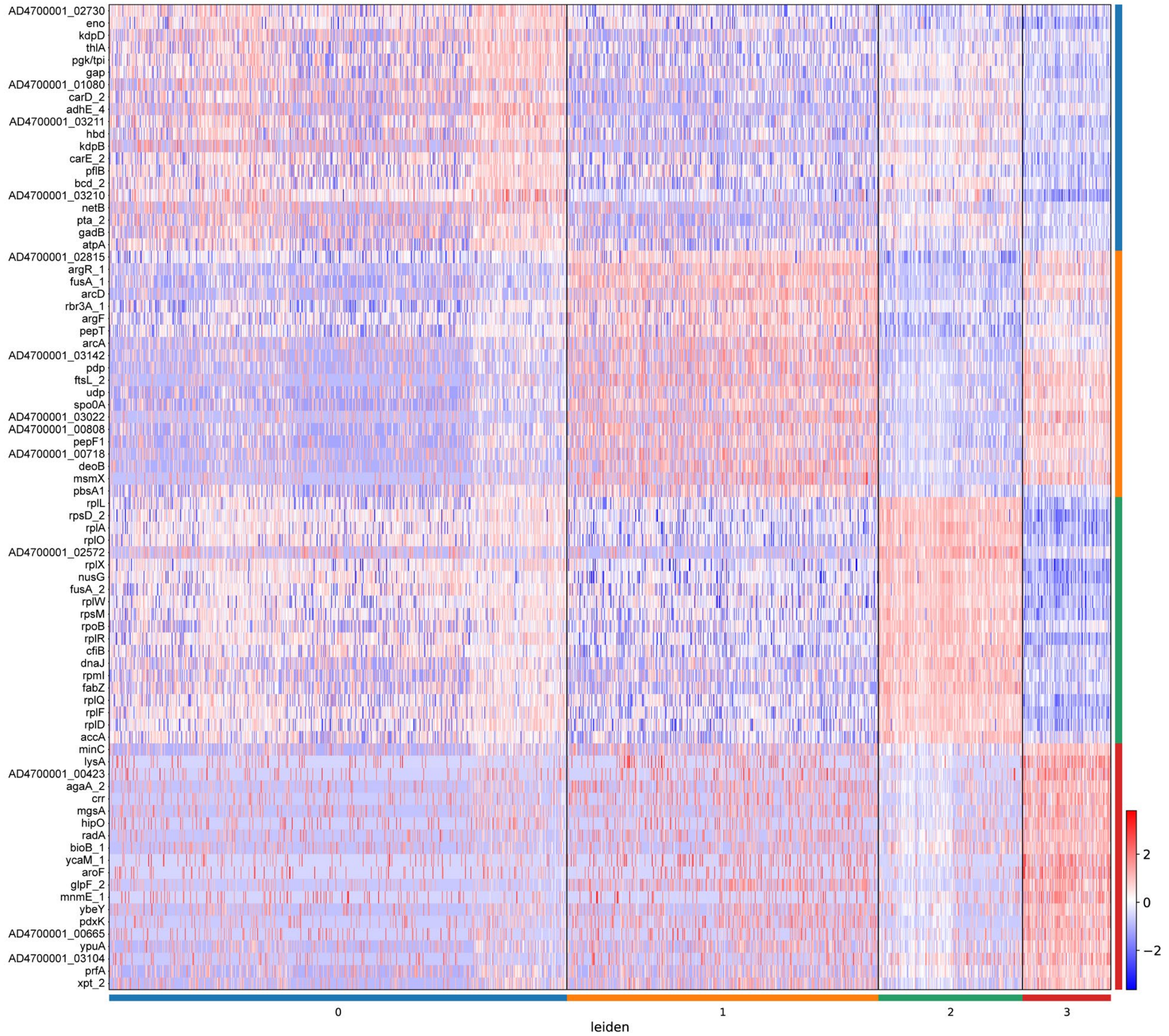

More information on gene names represented by gene locus-tags (for example AD4700001_02730) can be found in supplementary table 11 or in the genome assembly associated with this study

Figure S10: Heatmap of *C. perfringens* grown in BHI media with 4mM acetate from figure 4 including all marker gene names or genome locus tags – to accompany data in figure 4

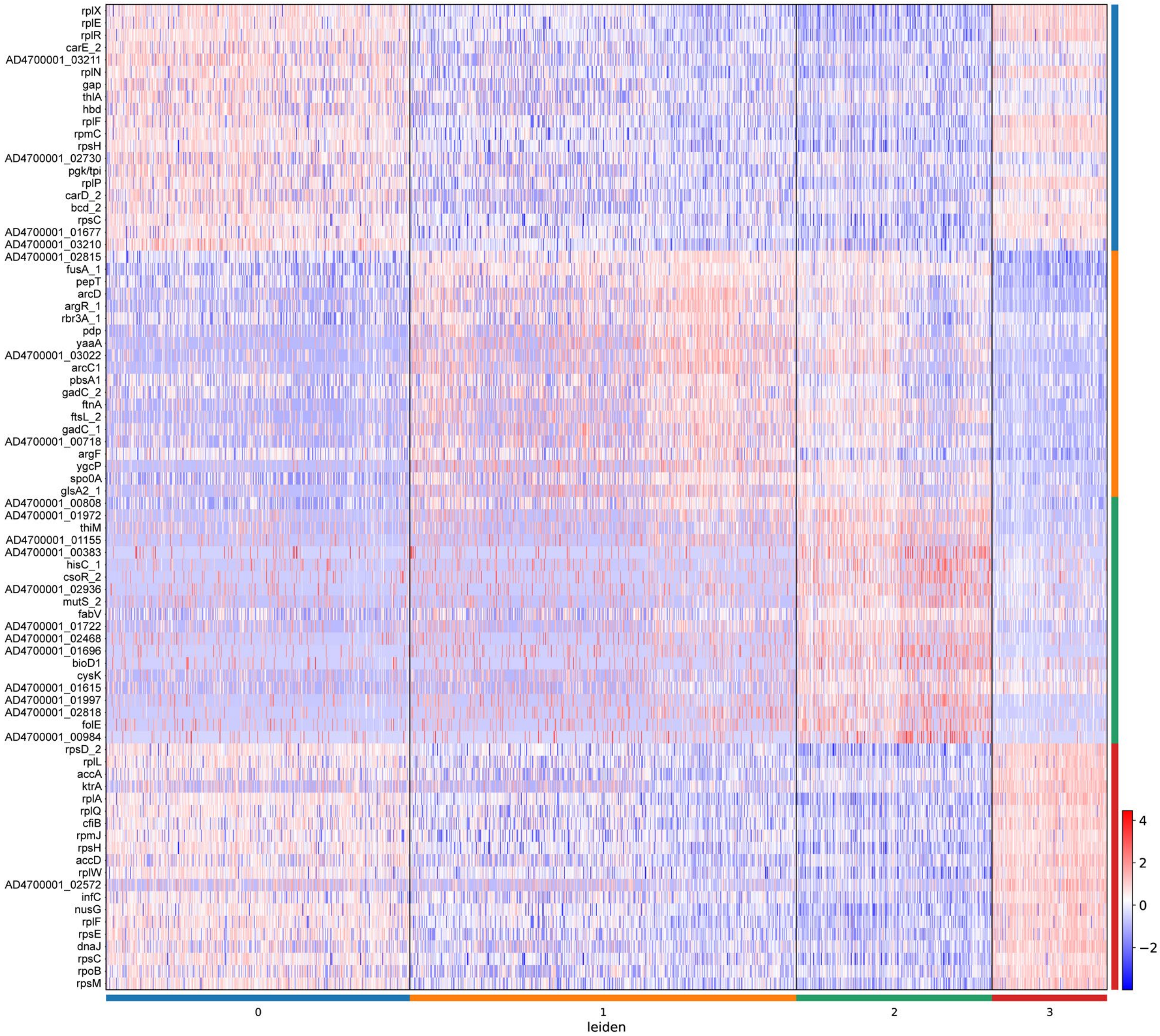

More information on gene names represented by gene locus-tags (for example AD4700001_02730) can be found in supplementary table 11 or in the genome assembly associated with this study

Figure S11: Presence of acetate in the culture media reduces the level of extracellular netB toxin

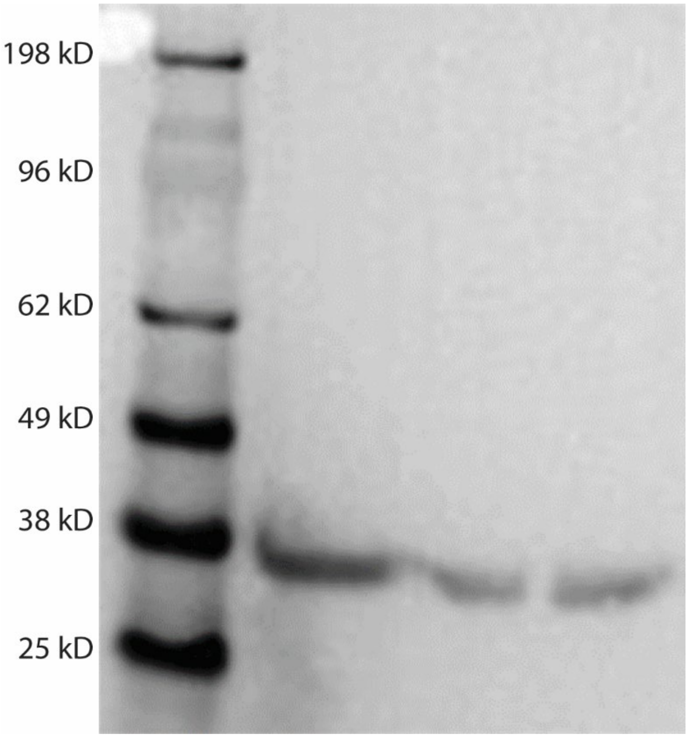

Western Blot analysis of netB protein found in the culture media of *Clostridium perfringens* cells grown in different conditions. Lane 1 was loaded with a molecular weight ladder (SeeBlue Plus2 pre-stained ladder - Novex). *C. perfringens* cultures were grown to the same OD (≈ 0.7-0.8) in unamended BHI media (lane 2), MRS media which contains ≈ 4mM Na-Acetate in standard recipe (lane 3), or BHI media to which 4mM Na-acetate was added (lane 4). Conditioned media was filtered through a 0.2uM filter and loaded in equal volume in each lane. A custom antibody for netB was used to detect netB near the expected protein size (33 Kd). Both media containing acetate had a reduction in extracellular netB compared to the non-acetate control. Toxin netB (33Kd) was detected using custom polyclonal rabbit antibodies (ProSci, Poway, CA) and the WesternBreeze rabbit chromogenic Western Blot kit (Invitrogen).

Figure S12: In Situ Hybridization protocol optimization reveals that addition of the cell wall degrading enzyme lysozyme helps increase probe signal

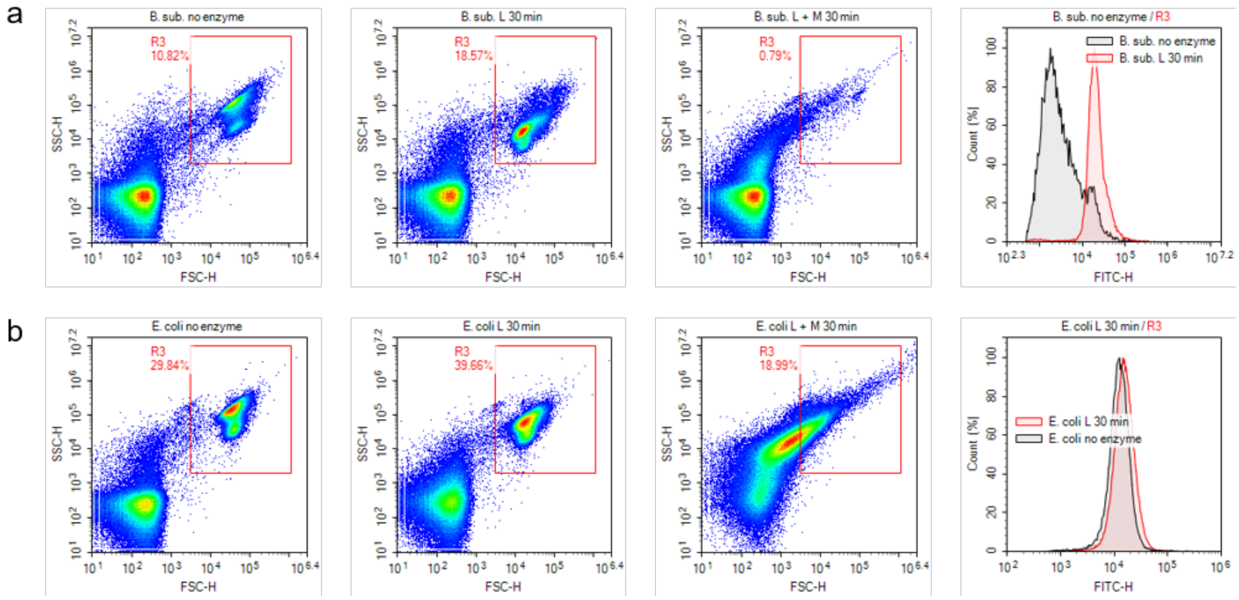

In situ hybridization conditions were optimized for increased probe penetration by measuring the fluorescent intensity of cells hybridized with fluorescently labeled rRNA probe Eub338 (Amann et al. 1990). Cells of either *B. subtilis* (panel **a**) or *E. coli* (panel **b**) were either not treated with cell wall degrading enzymes (left column), treated with lysozyme alone (middle column) or treated by a combination of lysozyme and mutanolysin. For both bacteria intact cell particles, boxed by the red rectangle, remain in similar morphological characteristics (FSC x SSC) with the addition of lysozyme treatment, but change dramatically by addition of mutanolysin, where cell size (FSC) decreases and an abundance of small particles appear. This is consistent with cellular lysis upon aggressive enzymatic treatment. As seen in the right-most column the addition of lysozyme increases the fluorescent intensity (FITC) of cell-sized particles, indicating that an increase in the binding of fluorescent probes. This increase is most notable for *B. subtilis* cells but is also observable to a lesser extent in *E. coli*.

Figure S13: Over 90% of cells are retained after ProBac-seq in-situ probe hybridization and washes

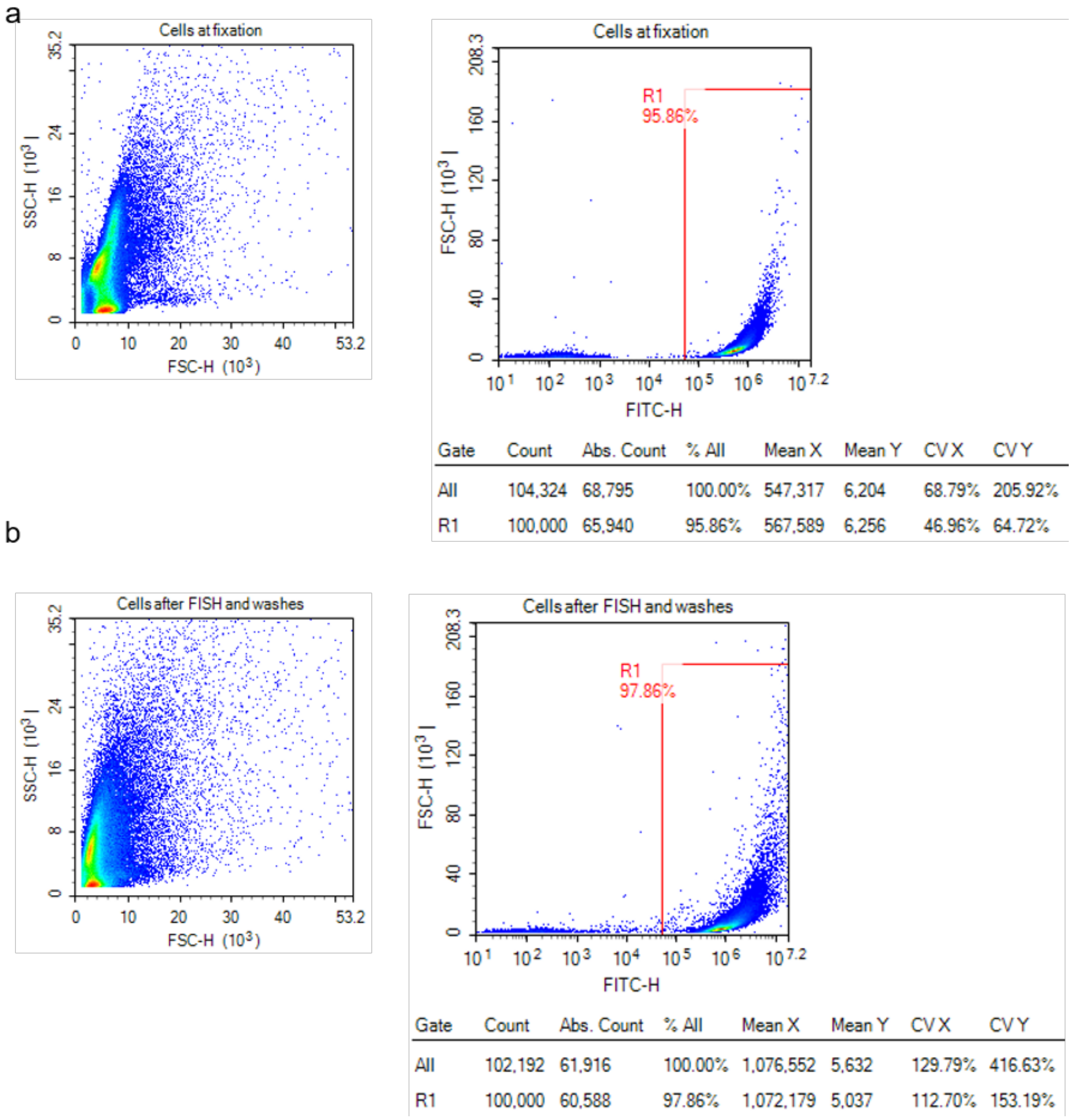

Gate	Count	Abs. Count	% All	Mean X	Mean Y	CV X	CV Y
All	104,324	68,795	100.00%	547,317	6,204	68.79%	205.92%
R1	100,000	65,940	95.86%	567,589	6,256	46.96%	64.72%

Gate	Count	Abs. Count	% All	Mean X	Mean Y	CV X	CV Y
All	102,192	61,916	100.00%	1,076,552	5,632	129.79%	416.63%
R1	100,000	60,588	97.86%	1,072,179	5,037	112.70%	153.19%

Flow cytometry was used to quantify the percentage of bacterial cells that are lost during probe hybridization and extensive washes used in the ProBac-seq protocol. **a.** Freshly fixed *B. subtilis* cells were labeled with syto-9 fluorescent dye as per manufacturers recommendation and analyzed in an Acea Quanteon flow cytometer (Agilent). Morphological characteristics (FSC x SSC) are shown on the left most panel. The fraction containing cell particles is stained by syto-9 and is gated by red threshold rectangle (right panel, R1 gate). Gated cells in R1 are automatically quantified by the instrument, which divides the number of gated events by the volume of sample analyzed (absolute count, tables below plots). **b.** After probe hybridization and all ProBac-seq washes were carried out cells were resuspended in the same volume as the original fixed suspension and again analyzed by cytometry using the same gates. In comparing the absolute number of cells in R1 region from experiment start to end over 90% of cells remain in the sample (60,588 / 65,940 x 100 = 91.88%)

Figure S14: scRNAseq of B subtilis using 10x UMIs instead of probe UMIs

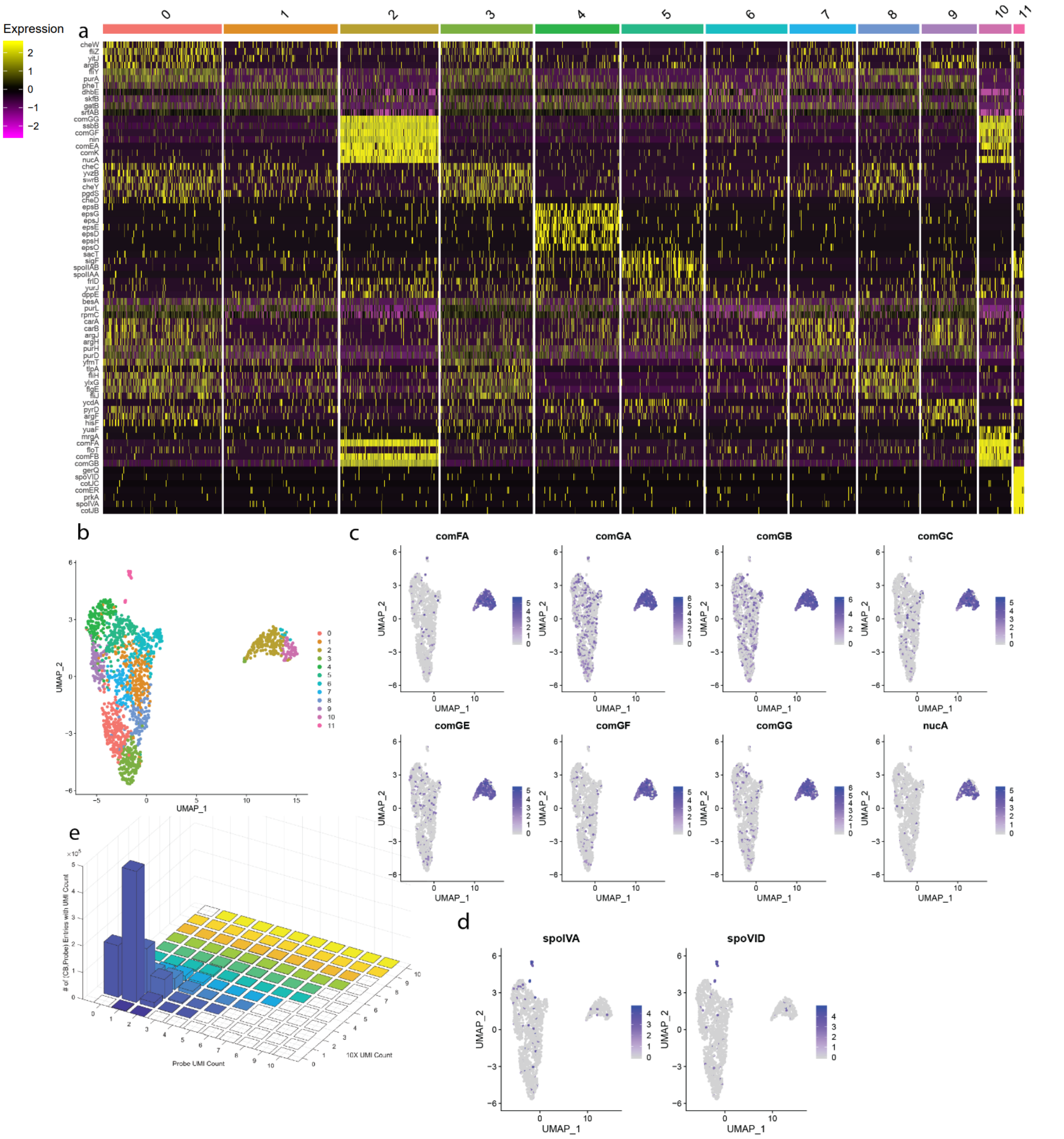

a. Heatmap of single cell gene expression **b.** UMAP projection of the 12 clusters **c.** Competence associated genes are predominantly expressed by cells in cluster 2. **d.** Sporulation associated genes are predominantly expressed by cells in cluster 11. **e.** Coincidence histogram of single-cell probe matrix constituted using Probe UMIs vs 10x UMIs (see Methods Section: Comparing Single-Cell Probe Expression Matrices Generated Using 10x vs Probe UMIs).

Figure S15: scRNAseq of E. coli in aerobic M9 culture using 10x UMIs instead of probe UMIs

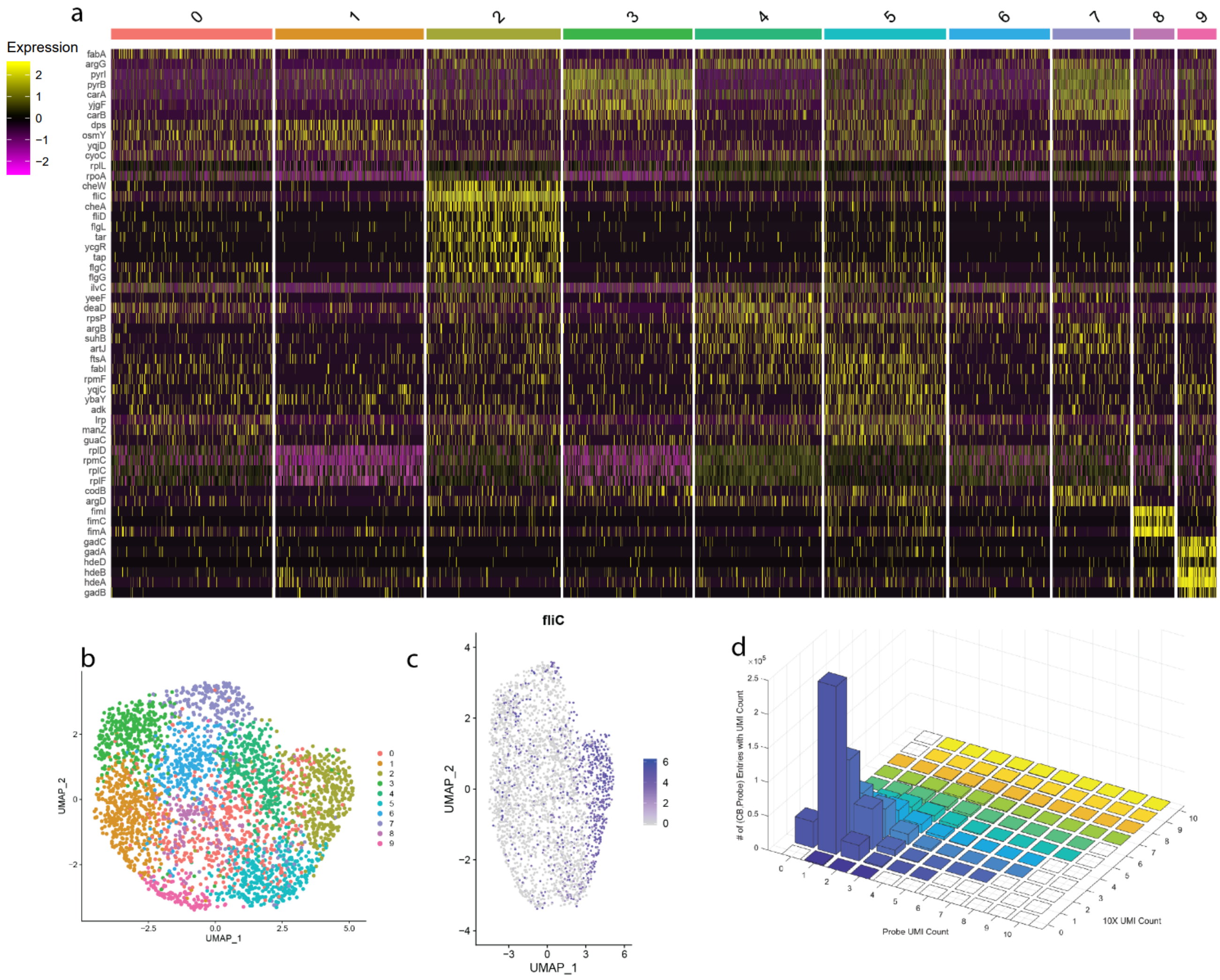

a. Heatmap of single cell gene expression (z-scores of log-transformed values) **b.** UMAP projection of the 10 clusters **c.** Flagellation associated genes are predominantly expressed by cells in cluster 2. **d.** Coincidence histogram of single-cell probe matrix constituted using Probe UMIs vs 10x UMIs (see Methods Section: Comparing Single-Cell Probe Expression Matrices Generated Using 10x vs Probe UMIs).

Figure S16: scRNA-seq of *B. subtilis* using bulk median instead of per-cell maximum probe counts for gene expression

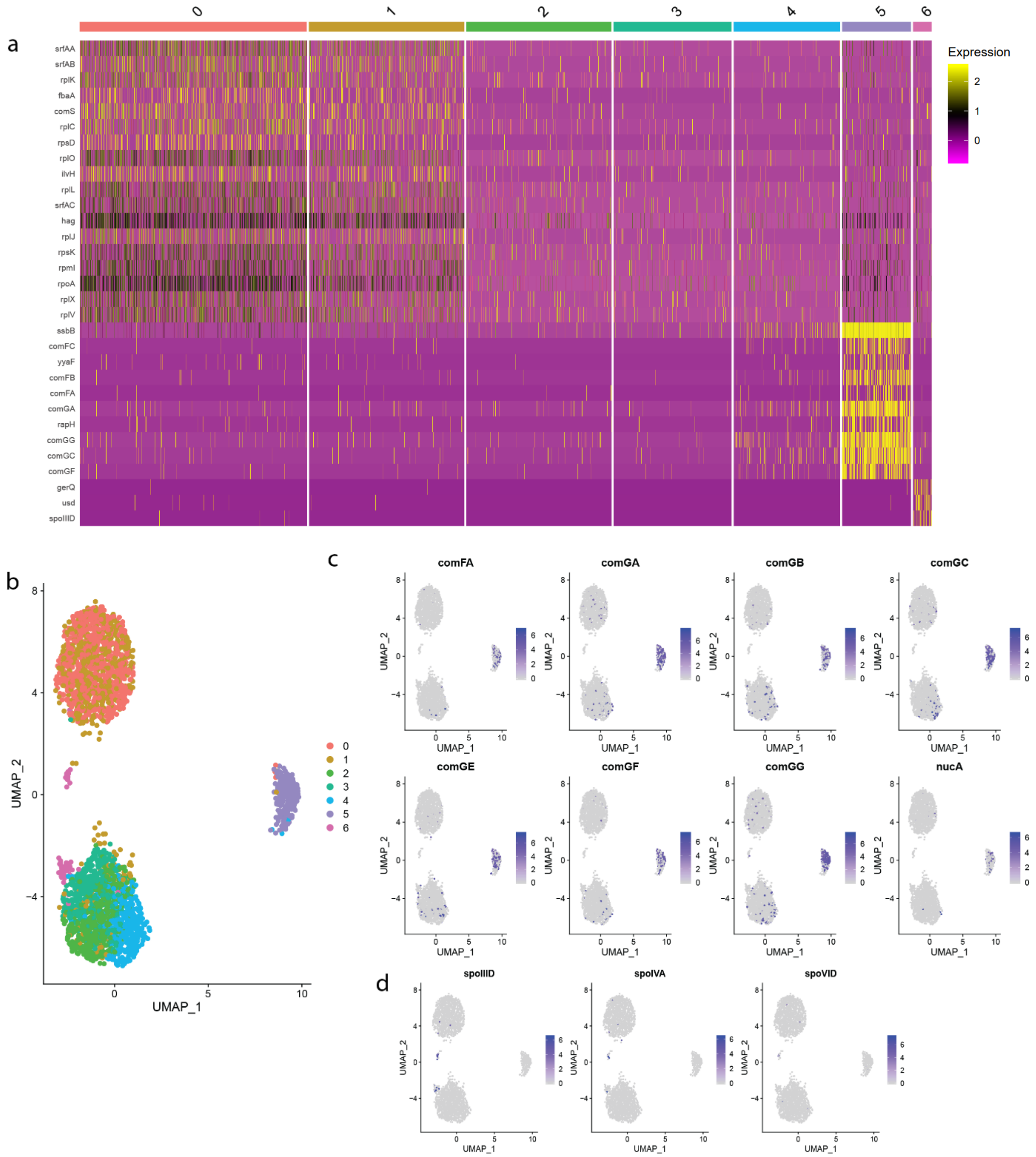

a. Heatmap of single cell gene expression (z-scores of log-transformed values) **b.** UMAP projection of the 7 clusters **c.** Competence associated genes are predominantly expressed by cells in cluster 5. **d.** Sporulation associated genes are predominantly expressed by cells in cluster 6.

Figure S17: scRNAseq of *E coli* cells in aerobic M9 media using bulk median instead of per-cell maximum probe counts for gene expression

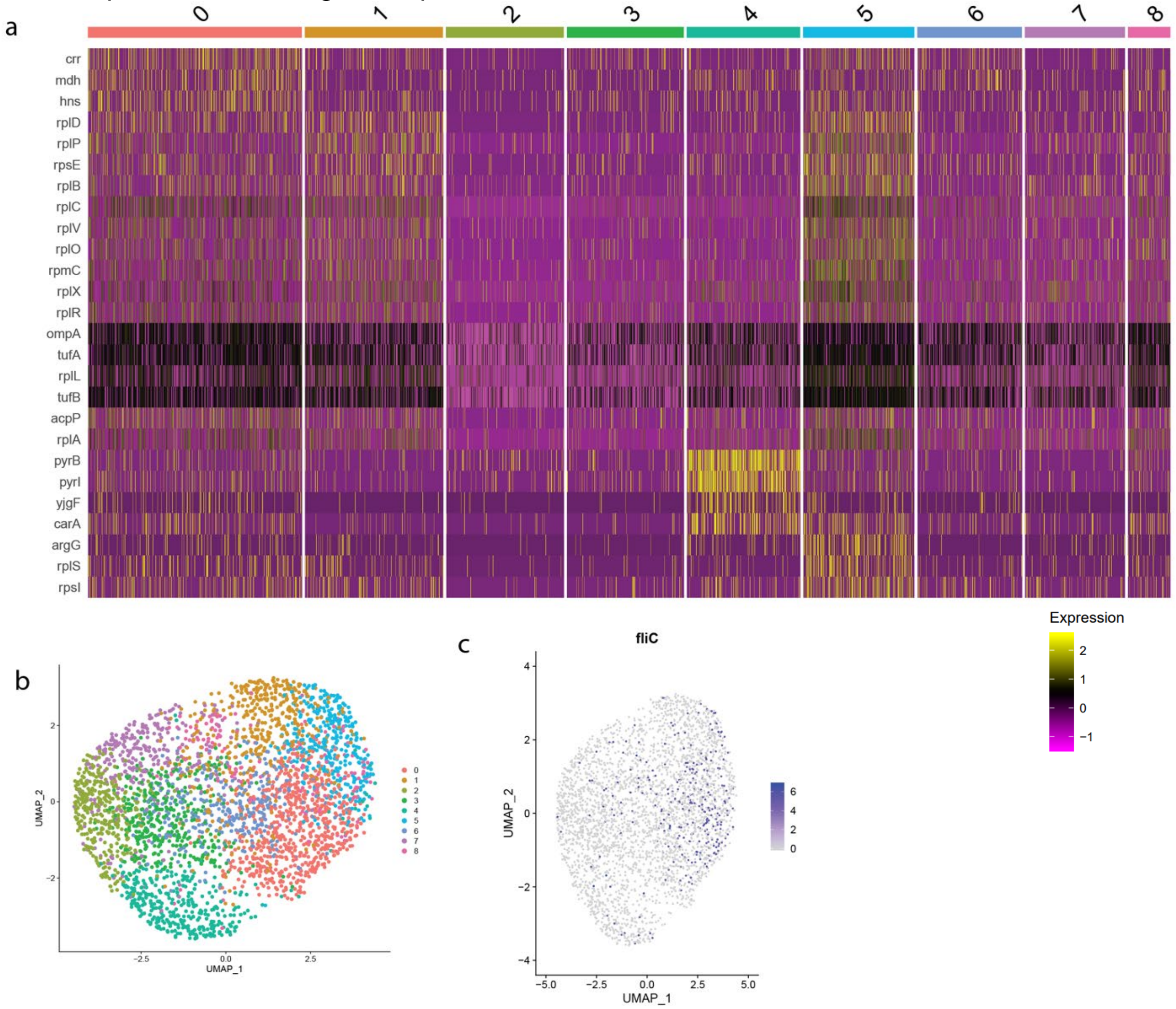

a. Heatmap of single cell gene expression (z-score of log-transformed values) **b.** UMAP projection of the 9 clusters **c.** Flagellation associated genes are predominantly expressed by cells in cluster 5.

Figure S18: Reproducibility of biological replicates grown in BHI condition

a

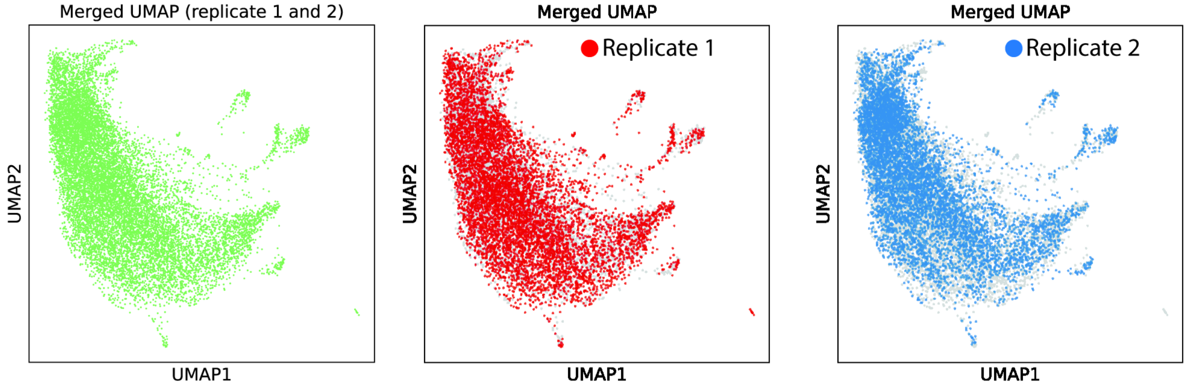

b

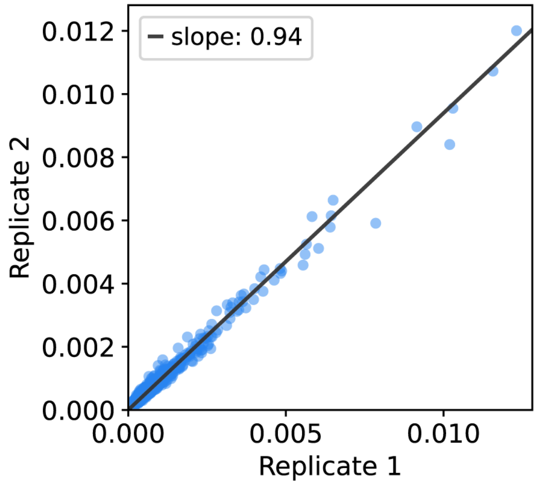

c

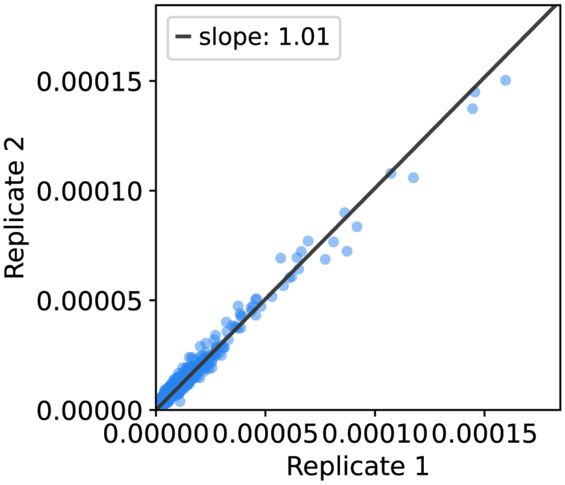

To assess the reproducibility of ProBac-seq, we compared the output produced by single cell analysis of two *C. perfringens* cultures grown separately in BHI medium without the addition of acetate (biological replicates). **a.** UMAP projections of all cells from the combined dataset is shown on the panel on the left, and the separated signal from replicate 1 and replicate 2 is shown on the middle and right panels, respectively **b.** scatter plot of mean frequencies of genes from the two replicates. The mean is computed across all resolved cells. r2=0.989 The figure is magnified to highlight lowly expressed genes and does not show two outliers with high expression values, rpsF and 16s rRNA, which are included for the fit and have coordinate values of (2.35e-2, 2.42e-2) and (1.69e-2, 1.38e-2), respectively. **c.** same plot as **b** showing variance of frequencies. r2=0.972. rpsF and 16s rRNA have coordinate values of (5.7e-4, 6.69-4) and (4.74e-4, 3.74-4), respectively.

Figure S19: Probe based transcriptomic measurement correlates strongly with traditional RNAseq transcriptomic measurement

a

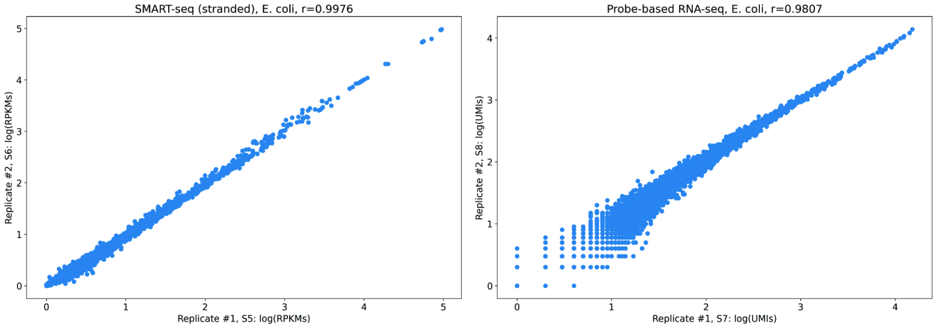

b

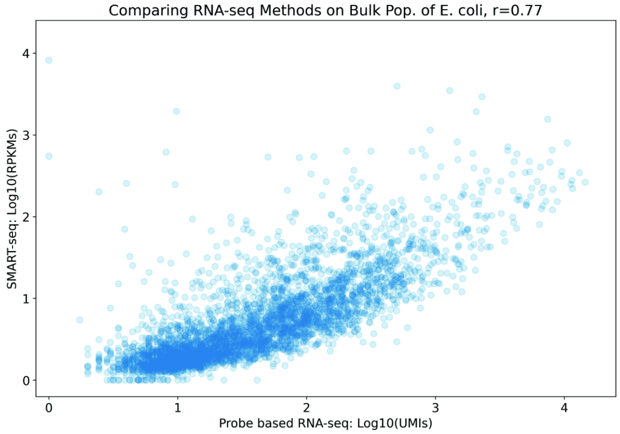

c

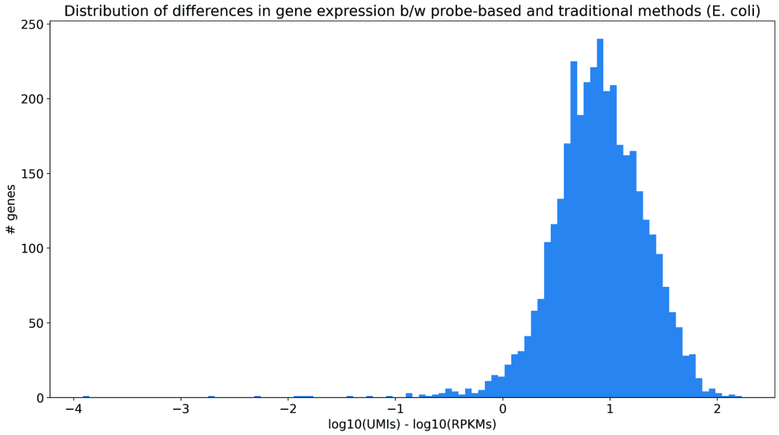

a. Gene expression of exponentially growing *E. coli* (str. MG1655) between technical replicates of different RNA-seq methods. Left) SMART-seq stranded RNA-seq (Takara). Right) Novel probe-based RNA seq (this paper). For both methods, libraries were prepared from a bulk population of cells (no single cell encapsulation). Reported *r* value is Pearson correlation coefficient. **b.** Pearson correlation (*r*) between methods, averaging the values obtained from the technical replicates. N=3579 genes total as probes were not designed for all genes captured by SMART-seq protocol. **c.** Distribution of differences between expression levels measured using the different protocols.

Figure S20: scRNAseq of *B subtilis* using in-droplet reverse transcription (RT) instead of in-droplet PCR identifies sporulation and competence populations

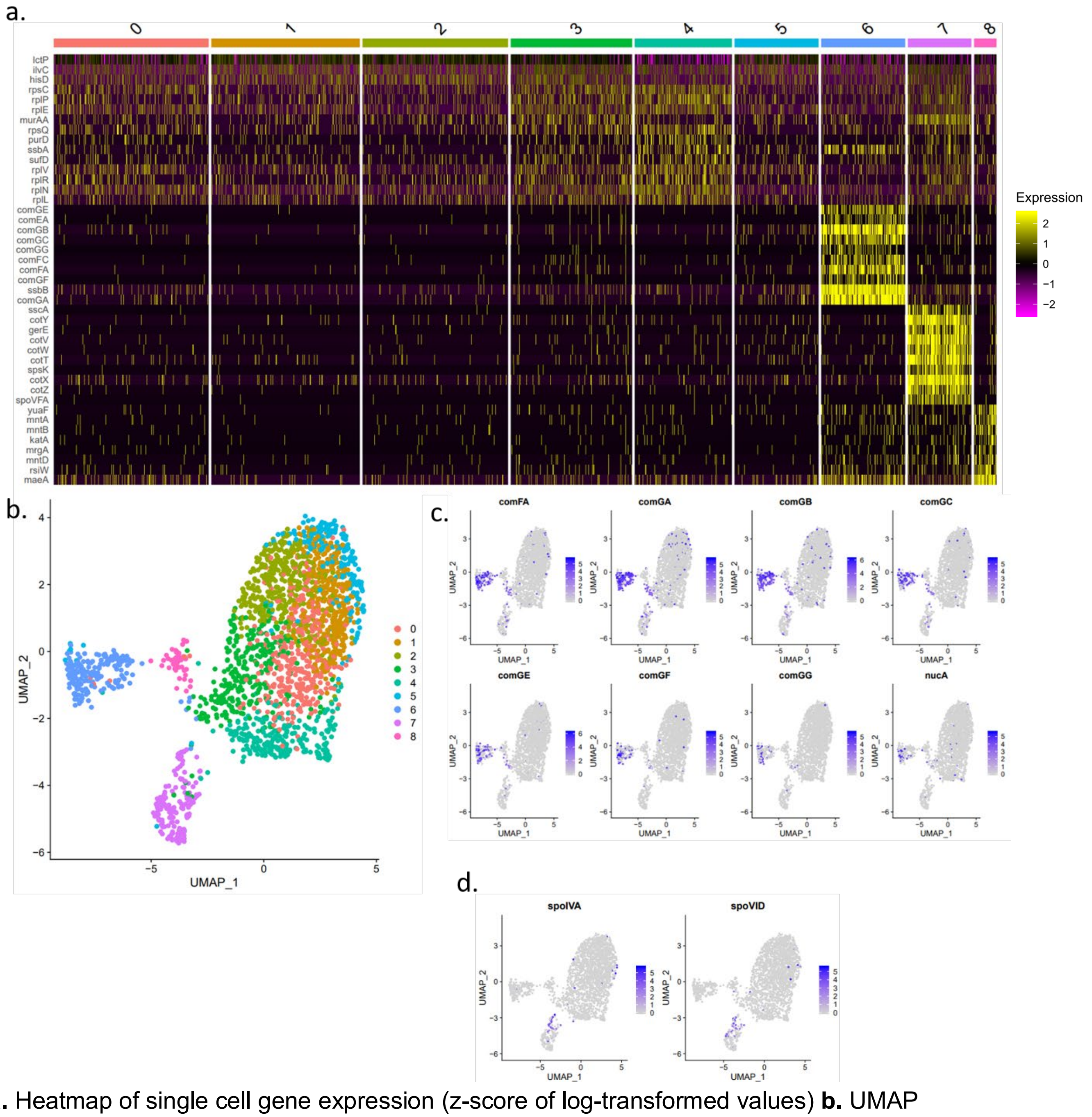

a. Heatmap of single cell gene expression (z-score of log-transformed values) **b.** UMAP projection of the 8 clusters **c.** Competence associated genes are predominantly expressed by cells in cluster 6. **d.** Sporulation associated genes are predominantly expressed by cells in cluster 7

Figure S21: *In situ* Hybridization optimization for *Clostridium perfringens*

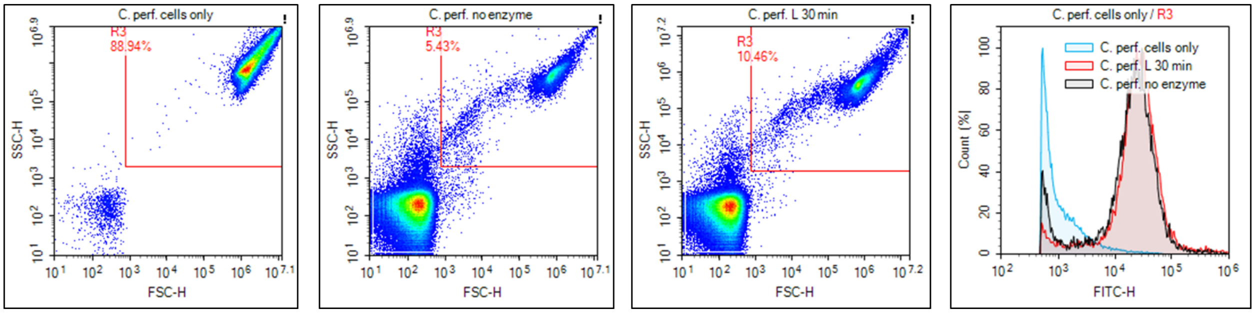

In situ hybridization conditions were optimized for increased probe penetration by measuring the fluorescent intensity of cells hybridized with fluorescently labeled rRNA probe Eub338 (Amann et al. 1990). Cells of *C. perfringens* were either not treated with probe (left panel), treatel with no cell wall degrading enzymes (second from left panel), or treated with lysozyme. Intact cell particles, boxed by the red rectangle, remain in similar morphological characteristics (FSC x SSC) with the addition of lysozyme treatment. As seen in the right-most column the addition of lysozyme slightly increases the fluorescent intensity (FITC) of cell-sized particles, indicating that an increase in the binding of fluorescent probes.

Supplementary Protocol 1: Constructing a Reference Genome

Here we describe how we constructed reference genomes tailored to the probesets used for each organism (see Methods Section Probeset Design). For a given organism, let P be the number of probes in the probeset, $\vec{s}_{dig} = AAGCTT$ be the $L_{dig} = 6$ base pair (bp) nucleotide sequence of the HindIII digestion site (see Supplementary Figure S1a), $\vec{s}_{ext} = TGCTCGAAGAAACTATG$ be the $L_{ext} = 17$ bp nucleotide sequence of the extender, $\vec{s}_{PCR} = TGTATGGACCGGGTCATC$ be the $L_{PCR} = 18$ bp nucleotide sequence of the PCR handle, and $\vec{p}_j$ be the L_j bp nucleotide sequence of the jth probe. Let $\vec{g}_j = [\vec{s}_{dig}, \vec{s}_{ext}, \vec{p}_j, \vec{s}_{PCR}]$ be the $L_{dig} + L_{ext} + L_j + L_{PCR}$ bp nucleotide sequence formed by flanking the jth probe by the HindIII digestion site, extender sequence, and PCR handle as shown in Figure 1a. We first constructed a FASTA file by concatenating all P of the $\vec{g}_j$ sequences. Next, we constructed a GTF file to index the FASTA. Typically, the GTF indexes genes in the FASTA by recording the location of the starting and ending bps of each gene. Since we have multiple probes per gene, and our FASTA is a concatenation of these probes, we therefore use the GTF to index probes instead. Specifically, we recorded that for the jth probe, the starting and ending bps of $\vec{g}_j$ in the FASTA are $\left(\sum_{i=1}^{j-1} L_{dig} + L_{ext} + L_i + L_{PCR}\right) + 1$ and $\left(\sum_{i=1}^{j} L_{dig} + L_{ext} + L_i + L_{PCR}\right)$ respectively. We use $\vec{g}_j$ as the definition of a probe instead of $\vec{p}_j$ for the purpose of alignment because sequenced reads should contain the flanking sequences anyways so including them minimizes spurious alignment of off-target reads. Lastly, we constructed a reference genome by using the *mkref* command in CellRanger (v3.1.0), supplying the generated FASTA and GTF files to the *--fasta* and *--genes* arguments respectively.

Supplementary Protocol 2: Creating FASTQ Files for CellRanger

Each read in the Illumina-sequenced FASTQ file contains the 10X CB, the 10X UMI, the Probe UMI as well as the mRNA sequence on a single line. This file therefore needs to be re-formatted to be compatible with CellRanger, so that the CB and UMI is stored in the R1 file and the mRNA sequence is stored in the R2 file. We also perform filtering at this stage so that only reads containing all the information needed by the CellRanger pipeline are retained. First, we discard reads whose length is less than or equal to 110 bp. Next, we aligned each read to $\vec{s}_{ext}$ using the *nwalign(glocal=true)* function in MATLAB (2019a), and discarded reads where the alignment score was <17. We search for the Extender sequence specifically, because for all probe designs the Extender sequence flanks the Probe UMI sequence (see Supplementary Figure S1c).

We create two sets of FASTQ files using reads retained after these filtering steps. In the first set of FASTQ files, we record the 10X CB and Probe UMI in R1, with the Probe UMI obtained by extracting 12 bps in either the 5' or 3' direction from the Extender sequence according to the probe design (see Supplementary Figure S1c). In the second set of FASTQ files, we record the 10X CB and 10X UMI in R1. For both sets of FASTQs, we trimmed all nucleotides 5' (3') of the Extender sequence if the Extender is 5' (3') of the binding region and record the result in R2. The two sets of FASTQs therefore have the same number of lines, identical R2 files and the same 10X CBs in the R1 files, and only differ in the UMIs recorded in the R1 files. We obtained single cell count matrices from these reformatted FASTQs by using the *count* function in CellRanger (v3.1.0) and supplying the custom made genome (described in Methods Section Constructing a Reference Genome) as the *--transcriptome* argument. Since CellRanger discards reads if QC scores for UMIs are too low, the same set of reads may not necessarily end up being used in constituting single-cell count matrices from the two sets of FASTQ files.

Supplementary Protocol 3: Comparing Single cell Probe Expression Matrices Generated Using 10X vs Probe UMIs

The output from CellRanger is a single cell matrix where each row, i, corresponds to one of N cells, and each column j corresponds to one of P probes. When the supplied FASTQ files have Probe (10X) UMIs recorded in R1, each entry in the single cell matrix, $c_{i,j}^{p}$ ($c_{i,j}^{p'}$) is the number of Probe (10X) UMIs in cell i corresponding to probe j. The Probe UMI remains fixed over multiple rounds of PCR and can therefore be treated as a classical UMI, while in our protocol, new 10X UMIs may be introduced on the same transcript at each PCR round. Therefore, $c_{i,j}^{p'}$ may not accurately record the number of transcripts. We used $c_{i,j}^{p}$ for all results presented in the main text of the paper. Since it may be more convenient to use 10X UMIs directly, we also quantified the extent to which $c_{i,j}^{p}$ differs from $c_{i,j}^{p'}$ by computing a joint distribution of count frequencies, $h_{k,l} = \sum_i \sum_j I_k\left(c_{i,j}^{p}\right) I_l\left(c_{i,j}^{p'}\right)$, where $I_s(t) = \delta_{s,t}$ (see histograms in Supplementary Figures S14-S15).
On the one hand, when $h_{k,l} > 0$, for $l > k$, there is over-counting; the sequencing depth is large enough to detect spurious 10X UMIs introduced in latter PCR rounds. On the other hand, when $h_{k,l} > 0$, for $l < k,$ there is under-counting; CellRanger detects that the same 10X UMI is coincidentally found on different probes, and discards all such reads. Since under-counting doesn't introduce spurious transcript counts and can be treated in the same vein as low sequencing depth or low capture efficiency, we do not consider it a confounder. The accuracy of a protocol relying only on 10X UMIs can therefore be quantified by the fraction of 10X UMIs corresponding to entries in $h_{k,l}$ with $l \leq k$:

$$Accuracy = \frac{\sum_k \sum_{l \leq k} l h_{k,l}}{\sum_k \sum_l l h_{k,l}}$$

For the *B. subtilis* sample, the accuracy of using 10X UMIs thus computed was only 41%. However, when we computed gene expression matrices using $c_{i,j}^{p'}$ (see Methods Section Single cell Gene Expression Matrices from Single cell Probe Expression Matrices below) and performed differential gene expression (see Methods Section Transcriptomic Analysis and Visualization below), we were still able to detect clusters corresponding to competence and sporulation (see e.g. clusters #2 and #11 respectively in Supplementary Figure S14 and Supplementary Table 9). Likewise, for the *E. coli* sample grown in minimal media, the accuracy was only 33%, but we were still able to detect clusters corresponding to the *fim* operon, cell motility and differential usage of carbamoyl phosphate (see e.g. clusters #8, #2 and #7 respectively in Supplementary Figure S15 and Supplementary Table 9). Taken together, this suggests that users of our technology may be able to use 10X UMIs directly i.e. $c_{i,j}^{p'}$. However, for the remainder of this study we restrict our attention to $c_{i,j}^{p}$.

Supplementary Protocol 4: Single cell Gene Expression Matrices from Single cell Probe Expression Matrices

To perform standard single cell analysis such as cell-similarity clustering and differential gene expression, we need to determine $c_{i,k}^{g}$, the number of transcripts of gene k in cell i, whereas the output from CellRanger is $c_{i,j}^{p}$. Let G_k be the indices of probes in the probeset corresponding to gene k. Since multiple probes may bind to the same transcript, setting $c_{i,k}^{g} = \sum_{j \in G_k} c_{i,j}^{p}$ may result in an over-estimate of transcript counts. To be conservative while also retaining as many UMIs as possible, we therefore picked $c_{i,k}^{g} = c_{i,j}^{p}$ which lower bounds gene expression. This choice resulted in 66%, 79% and 81% of the UMIs in c^p being retained in c^g, accounting for 65%, 77% and 81% of the total reads, for the *B. subtilis*, *E. coli* in minimal media and *E. coli* in LB media samples respectively. That different probes may be selected to represent the same gene in different cells is not material, since probes are selected proportional to their binding affinity. However, this choice introduces a potential bias since the gene expression tabulated in c^g will tend to be higher for genes with more corresponding probes in the probeset, i.e. larger $|G_k|$. As a sanity check, we also tabulated single cell gene expression matrices using $c_{i,k}^{g} = c_{i,k^*}^{p}$, $k^* = \underset{j \in G_k}{\operatorname{argmedian}} \sum_i c_{i,j}^{p}$ i.e. using a fixed probe across all cells for each gene corresponding to the probe with the median bulk expression. This choice only resulted in 10%, 18% and 17% of the UMIs in c^p being retained in c^g, for the *B. subtilis*, *E. coli* in minimal media and *E. coli* in LB media samples respectively. Despite the decreased transcriptional resolution, we are able to still detect clusters corresponding to the results presented in the main text (see Supplementary Figures S16-S17 and Supplementary Table 10). We therefore defer investigation of more sophisticated methods of combining probe counts to form a single cell gene expression matrix to subsequent work.

Supplementary Protocol 5: Removal of spurious UMI

The *C. perfringens* experiments were done with probes that did not include UMI #2 (which is referred to as Probe UMI in the main text). Therefore, we used the 10X UMIs to call individual transcripts in these experiments. However, spurious 10X UMIs can be incorporated during the 6 cycles of in-droplet PCR, and result in overcounting of the number of detected transcripts. The number of spurious UMIs increases with the depth of sequencing. Here, we describe how we computationally remove the spurious UMIs by using the number of reads associated with each UMI.

Spurious UMIs are incorporated during the in-gem PCR because the reverse strand can be copied only after a new 10X primer—with a different sequence for UMI #1—hybridizes to the poly-A sequence. We refer to all UMIs incorporated in this manner as spurious UMIs. As an example, let us imagine the first three cycles of a *perfect* in-gem PCR. After the first cycle, there will be two strands with the same UMI. After the second cycle, however, there will be three strands with the same UMI and one strand with a different UMI, as this strand had to be primed off a new 10X primer. After the third cycle, there will be 4 strands with the *original* UMI, 2 strands with the *previously incorporated* UMIs, and the other 2 strands, each with a different *newly incorporated* UMI. Continuing with the pattern that copying forward strands increments the number of reads associated with their UMIs by one while copying each reverse strands introduce a new UMI, by the time we finish the 6th cycle, there will be $2^6 = 64$ strands and $2^5 = 32$ UMIs, where the original UMI has 7 reads, another UMI has 5 reads, 2 UMIs have 4 reads, and so on. If there were no such proliferation, we would have a single, original UMI with 64 reads. We will use the fact that spurious UMIs have fewer reads than the original UMI to identify and remove them.

To remove the spurious UMIs, we first obtained the number of reads of each UMI (which we will refer to as UMI count interchangeably) per cell barcode by scanning every read in the BAM file. We only accounted for reads each with a mapped probe, an error-corrected cell barcode, and an error-corrected UMI by requiring "GN", "CB", and "UB" tags be present for a given read. We kept UMIs with at most 8 reads (corresponding to 99.98% and 99.99% of UMIs in BHI and BHI +acetate conditions, respectively), aggregated the UMI counts associated with the top 2000 cell barcodes with the greatest number of total reads, and generated a normalized histogram of UMI counts shown in Figure S7.a. We also divided the top 2000 cell barcodes into 10 bins of 200 cell barcodes each and generated normalized histograms for each bin.

To obtain the expected number of reads of the spurious of UMIs after two rounds of noisy PCRs and a subsampling, we used simulations. We simulated the noisy amplification of a single transcript through in-droplet PCR by deciding whether to copy each existing read via a Bernoulli draw with a success probability of 0.8. We introduced a new UMI whenever a reverse strand was copied and kept track of the number of reads associated with each UMI (referred to as UMI count). We denote the UMI counts as $\{N_i\}_{i=1}^{K}$ where K is the total number of distinct UMI incorporated. As explained above, the greatest possible K is 32 and the greatest possible N_i is 7. We then simulated the out-of-droplet PCR by drawing an amplification increment N_i for each UMI count from binomial distribution where the number of trials is current N_i and success probability is 0.8, and iterated this process 16 times, each time with an updated number of trial $N_i \leftarrow N_i + \widetilde{N}_i$. Assuming independent amplification processes, we repeated the two PCR simulations 100000 times to generate enough samples of UMI counts so the histogram of aggregated counts, shown in Figure S7. b, is statistically invariant. In a real experiment, only a small fraction of amplified reads N_i is sequenced. We modeled this subsampling process as a Poisson draw with a mean rate $\lambda_i = N_i \times r$, where r is a subsampling factor. We optimized r by minimizing Jensen-Shannon divergence between a normalized histogram of the subsampled

UMI counts, aggregated over the 100000 trials, and the normalized data histogram from the BAM file. As a negative control, we assumed in-droplet PCR amplifies the original UMI count to 64 and simulated the out-of-droplet PCR and the subsampling process as described above. We also optimized r against the normalized histogram from each of the 200 cell barcode bins. Since a particular optimization run relies on stochastic samples, we repeated the optimization run 50 times. Optimization results and normalized histogram of UMI counts subsampled using the mean of optimal values of r (2.77e-5 for BHI and 2.33e-5 BHI + acetate) are shown in Figure S7. c-d.

After subsampling with the optimal value of r, spurious UMIs often resulted in zero reads since their Poisson rate $\lambda_i < 1$. The original UMI, however, is likely to yield a non-zero read because it had the greatest number of chances to get copied (i.e., 6 times). We thus calculated the mean of the number of UMIs, over 50×100000 trials, originating from a single transcript with a non-zero UMI count after subsampling and used this value as a normalization factor to convert the observed number of UMIs from data to the actual number of UMIs. We repeated the subsampling Poisson draws 50 times for each trial to minimize sampling noise. Figure S7. e-f shows the histogram of the number of UMIs from 50×100000 trials. Importantly, because the optimal value of r did not change across the cells in both data sets in Figure S7. c-d, we applied the same normalization factor, 7.49 and 6.65, to all the cells in BHI condition data and BHI + acetate condition data, respectively. Because all downstream analysis of the count matrices is performed after normalizing the transcript counts in each cell by the total number of transcripts in that cell, the normalization introduced here has no bearing on the downstream analysis, rather it is only used for reporting the number of transcripts detected in each cell in the *C. perfringens* experiments.

Supplementary Protocol 6: Cell Calling

In-situ hybridization poses a unique challenge for cell calling because barcoded cDNA can originate not only from mRNA:probe hybridized complex but also from unwashed probes as well as probes bound to cell-free transcripts released from lysed cells and present as a background in all droplets. The artifactual signal from unwashed probes is unique to our method and not seen in conventional single-cell RNA sequencing methods, which do not use capture probes.

Here, we describe a statistical model we developed to robustly distinguish real cells from these "ambient" barcodes defined as the cell barcodes that originate solely from the unwashed probes and/or probes bound to cell-free transcripts.

We first collapsed probe expression matrix c^p, normalized using the factor identified from the previous section, into gene expression matrix c^g by picking $c^g_{i,k} = \max_{j \in G_k} c^p_{i,j}$ for gene k of cell barcode i as done in "Single cell Gene Expression Matrices from Single cell Probe Expression Matrices" section of Method. We ranked the cell barcodes in decreasing order of total count, $t_i = \sum_k c^g_{i,k}$, which is the total number of normalized transcripts in cell i. We obtained a cell barcode rank curve by plotting rank vs. total count as a log-log plot. Following Lun et. al. (EmptyDrops[1]), we set a lower threshold T and selected cell barcodes with $t_i < T$ as the ambient barcodes, assuming that the cell barcodes that have a low total count are the ambient barcodes. We also set an upper threshold U and defined cell barcodes with $t_i > U$ as real cells, assuming that any cell barcodes with a sufficiently large total count should represent a cell-containing droplet. We chose T and U such that cells above the top of the gradual drop-off region and below the start of the steep drop-off of the barcode-rank plot were selected respectively as shown in Figure S8. a and S8. c. The cell barcodes with $T \leq t_i \leq U$ are marked as putative cells.

To account for the differences in the total count across the cell barcodes, we computed the normalized counts of the genes for each cell barcode, defining a frequency matrix $\tilde{c}^g$ where $\tilde{c}^g_{i,k} = c^g_{i,k}/t_i$. We then calculated the mean and variance of the frequencies for each gene across both the real cells and the ambient barcodes. We observed that the variance scales linearly with the mean for the ambient barcodes whereas it increases faster than linearly for the real cells (Figure S8. b and S8. d). We ran a linear regression to find the proportionality constant between the mean and variance of the frequencies of the genes in the ambient barcodes.

To construct a null model for a given cell barcode with a total count t_i, we first multiplied the mean frequency of each gene of the ambient barcodes by t_i to get an expected mean count $\mathrm{Mean}[c^g_{i,k}]$ for each gene. To obtain the expected variance of each gene, we first computed the variance of the frequency from the mean frequency using the relationship, $\mathrm{Var}[\tilde{c}^g_k] = \alpha \times \mathrm{Mean}[\tilde{c}^g_k]$. We then converted the variance of frequency to a variance of count by scaling with total count t_i as follows,

$$\mathrm{Var}[c^g_{i,k}] = \mathrm{Var}[t_i \times \tilde{c}_{kg}] = t_i^2 \times \mathrm{Var}[\tilde{c}^g_k] = t_i^2 \times (\alpha \times \mathrm{Mean}[\tilde{c}^g_k])$$

Next, we constructed a negative binomial distribution where, given a mean count $\mathrm{Mean}[c^g_{i,k}]$, a dispersion parameter $\phi_{i,k}$ was set to give the observed value of $\mathrm{Var}[c^g_{i,k}]$ (i.e., $\phi_{i,k} = \frac{\mathrm{Mean}[c^g_{i,k}]^2}{\mathrm{Var}[c^g_{i,k}]^2 - \mathrm{Mean}[c^g_{i,k}]}$). The mean and variance ratio of count for cell barcode i, which is $t_i \times \alpha$ in our setup, should be greater than one for us to invoke a negative binomial distribution. Therefore, we

excluded ambient barcodes with $t_i < \frac{1}{\alpha}$. Taken together across each gene, this gave us a null model for a cell barcode's count profile.

Finally, we computed the log-likelihood L_i^{obs} that the observed counts in a cell barcode i come from the null model as follows,

$$L_i^{obs} = \sum_{k \in \{k\prime :\, \tilde{c}_{i,k\prime}^{g} \neq 0\}} \mathrm{logpmf}_{\mathrm{n.b.}}\left(c_{i,k}^{g};\, \mathrm{Mean}\left[c_{i,k}^{g}\right],\, \phi_{i,k}\right)$$

where $\mathrm{logpmf}_{\mathrm{n.b.}}$. is the logarithm of probability mass function of a negative binomial distribution, and we ignored genes that had zero frequency in the ambient barcodes, which were ~1% of total genes. We followed a Monte Carlo approach to compute a p-value for the cell barcode i {Lun et. al. (EmptyDrops)[1]}. We generated R count vectors by sampling from negative binomial distribution with $\mathrm{Mean}\left[c_{i,k}^{g}\right]$ and dispersion parameter $\phi_{i,k}$ across each gene k and evaluated their log-likelihood L_i^{sim}. The p-value of a cell barcode i was computed as $P_i = \frac{R_{sim}+1}{R+1}$ where R_{sim} is the number of times $L_i^{sim} \leq L_i^{obs}$ and one is added to avoid zero p-values for multiple testing. We use $R = 1000$ in all simulations.

The histogram of p-values for the real cells, the putative cells, and the ambient barcodes are shown in Figure. S8. e. The ambient barcodes are used to generate the null hypothesis. Although the distribution of p-values of the ambient barcodes was not uniform, the p-values of the real cells were $\frac{1}{1001}$, the smallest value permitted in our computation. The lack of p-values of ambient barcodes near zero and one may be attributed to excluding ambient barcodes with $t_i < 1$. The distribution of p -values of the putative cells had its peak close to zero and continuously decreased to near zero value. We used a conservative false discovery rate (FDR) threshold of 0.2% when performing multiple testing correction with Benjamini-Hochberg method.

Supplemental note 1: Additional biology related to Figure 2 and Figure S2

For *B. subtilis* in minimal media, in addition to the competent and sporulating cell subpopulations discussed in the main text, we observe two other clearly distinguishable cell groups indicative of distinct biological states. The largest group of cells (comprising clusters 1, 2, 3, 5, and part of cluster 7) upregulate *dhbA, dhbB, dhbC, dhbE,* and *dhbF* (the 5 enzymes implicated in the biosynthesis of bacillibactin) as well as *yusV*, an ABC transporter of bacillibactin. In addition, at least 3 out of the 5 clusters within the subpopulation showed upregulation of genes involved in thiamine biosynthesis (*tenA, tenI, thiD, thiG, thiF, thiS, thiO*; gene set enrichment = 16.85, FDR = 2.79E-05), amino acid biosynthesis (fold enrichment = 5.23, FDR = 1.48E-07), amino acid activation (*thrS, gatB, ileS, aspS*; fold enrichment = 6.22, FDR = 3.46E-02), surfactin biosynthesis (*srfAA, srfAB, srfAC, srfAD*), "de novo" IMP biosynthesis (gene set fold enrichment = 25.79, FDR = 9.10E-09), purine biosynthesis (*purB, purC, purD, purQ, purH, purF, purL, purK, purM, purS*; gene set fold enrichment = 21.66, FDR = 1.83E-02), pyrimidine biosynthesis (*pyrAA, pyrK, pyrF, pyrH;* gene set enrichment = 9.63, FDR = 2.59E-02), chorismate biosynthesis (*aroA, aroB, aroC, aroH, aroE*; gene set fold enrichment = 18.05, FDR = 4.60E-03), cell motility (*hag, fliY, fliD, fliH, flhP*; gene set enrichment = 6.19, FDR = 1.44E-02), sulfate assimilation (*cysI, cysH, cysC, cysJ;* gene set enrichment = 18.05; FDR = 4.56E-03), and biotin biosynthesis (*bioA, bioB, bioD, bioF, bioW*; gene set fold enrichment = 18.05, FDR = 4.65E-03). Altogether, this subpopulation appears to represent the most metabolically active cell state within the total population, an observation further supported by the fact that genes encoding ribosomal components & translation machinery were also enriched (*rpmI, rplU, rplR, rpsM, rpsE, rpsI, rpsN, rpsD, rpoB, rplV, rplX, rplB, rpmA, rpsS, rplS, rpsP, rplL, rpmD, rpmC*; "translation" gene set fold enrichment = 7.81, FDR = 3.07E-12). Cell cluster #2, part of the subpopulation, may represent the gateway into sporulation as early sporulation factors *sigF, spooA,* and *spoIIAB* are all uniquely upregulated and cells are projected in close proximity with cluster 9 after dimensional reduction. In addition, these cells downregulate genes implicated in motility (*hag, fliD, fliG, fliY, fliK, fliM, flgK, flgL*) in stark contrast to other cells within the subpopulation. Furthermore, cells within cluster #5 are distinguished from others in the "metabolically active" subpopulation by expression of genes related to arginine biosynthesis via ornithine. L-arginine is used by cells for protein synthesis as well as for production of polyamines such as putrescine and spermidine; compounds which have functions in nucleic acid binding, protein activation, and membrane stabilization due to their polycationic state. The 4th distinct cell state detected in *B. subtilis*, only slightly less in size compared to the "metabolically active" cell group (45% of total population), was characterized by lower expression of many of the genes upregulated in the "metabolically active" subpopulation. Uniquely, cells in cluster 0 (930 cells, 76% of the subpopulation) were found to overexpress *sdpB* (Log_2FC = 0.68, adjusted p-value = 4.7E-4). Under starvation conditions, *B. subtilis* produces an antimicrobial peptide through the *sdpABC* operon that is active against its own species, a tactic used by the cell to increase its own chance of survival and delay commitment to sporulation. While the *sdpC* gene encodes the 42AA toxic peptide, *sdpA* & *sdpB* are required for maturation of the peptide into its active form prior to secretion. *sdpB* is a multipass membrane protein which affects the toxicity of the final SDP peptide without being involved in signal peptide cleavage, disulfide bond formation, or secretion. The *sdpABC* operon was found expressed in cells within each of the four subpopulations, however, it is only within cluster #0 that sdpB was found to be significantly upregulated. This finding, along with the overall reduction in transcript load as compared to the "metabolically active" subpopulation, suggests a more dormant biological state in which cells appear to be facing a nutritional strain but have not yet committed to an alternative developmental program. This hypothesis is supported by the fact that cells in cluster 7 are split between the two largest subpopulations in the UMAP projection as well as by the clustering of a few cells associated with competence (part of cluster 8) near this dormant subpopulation – possibly suggesting a developmental transition between the two states. Please note that not all genes discussed will be

displayed as marker genes in all figures. This is due to the fact that marker genes are agnostically selected by the computational pipeline and is limited to only a few genes per cluster, excluding many genes from being displayed. To view the full set of genes that are significantly differentially regulated in any given experiment please refer to the appropriate supplementary DGE table.

Supplemental note 2: Benchmarking ProBac-Seq probe based transcriptomics to traditional RNAseq

To see if ProBac-seq transcriptome measurements are consistent with traditional methods we benchmarked probe-based data with data obtained from cells grown in the same conditions but analyzed by RNA-seq (Methods). We first compared a *B. subtilis* culture that was fixed in formaldehyde and split into two aliquots - one treated with probes and subjected to ProBac-seq hybridization and washes and the other in which RNA was extracted for RNA-seq. In this comparison (Figure 1e) we observe a Pearson correlation of $R^2 = 0.55$, which is consistent with correlations observed in the past for comparisons of gene expression microarrays and RNAseq data. While this finding demonstrates the method's ability to reproduce transcriptome measurements, it does not directly address the question of whether addition of formaldehyde is in itself interfering with the method's ability to report on the true transcriptomic state of the cells, since both samples were initially fixed with formaldehyde, a procedure that is not routinely done in traditional bacterial RNA-seq protocols. To account for the ability of formaldehyde to preserve transcriptomic signatures and to offer a more direct comparison of ProBac-seq and traditional RNAseq we performed an additional experiment in which ProBac-seq probe-signal from formalin-fixed *E. coli* cells was compared to a bulk RNA-seq of the same sample that was instead preserved in bacterial RNAprotect reagent (Qiagen), a common preservation used for bacterial RNA-seq in the literature. In this new data (Supplementary Figure S19), the correlation increases to 0.77. Furthermore, formaldehyde or other stress pathways are not differentially expressed in the formaldehyde-fixed ProBac-seq samples, suggesting that fixation happens before transcriptional responses can occur. This correlation is as good as the best correlations reported in comparisons of microarray vs RNA-seq in the literature and should re-assure that the data obtained faithfully reports on the transcriptional state of the cells. Interestingly, comparing bulk RNA-seq with or without formaldehyde suggests that formaldehyde fixation may introduce biases to traditional RNA-seq that are not affecting probed-based formaldehyde-fixed samples. One possible explanation is that crosslinks have to be reversed in RNA-seq of formaldehyde fixed samples, whereas the RNA remains intact in FISH-type approaches. Reversal of crosslinked RNA may cause degradation, shearing or loss of RNA, which may have negative implications for formaldehyde in traditional RNAseq techniques that rely on uncrosslinked RNA. Taken together, these experiments demonstrate that ProBac-seq fixation protocol and transcriptomic analysis is able to preserve and report transcript levels in fixed cells.

Supplemental note 3: Additional biology related to Figure 3a and Figure S6

Single cell transcriptomes of *E. coli* grown in M9 were clustered into ten groups. In the largest group of cells (cluster #0, 514 cells, 15.5% of total), ribosomal genes (*rpl operon, rpsC, rpsD, rpmC, rpmD*) as well as *deaD* and *priB* are amongst the most upregulated compared to the rest of the cell population. *deaD* ($log_2FC = 0.7$, adjusted p-value = 2.3E-11) is a DEAD-box RNA helicase involved in ribosome biogenesis, translation initiation, and mRNA degradation at low temperatures - forming a cold-shock degradosome with RNase E. Kuchina et al. also identified a subpopulation of *E. coli* differentially expressing *deaD*, along with other cold shock proteins, in

their scRNA-seq experiments and hypothesized the cluster was an artifact of sample preparation. Since our protocol fixes cells directly in culture, we find it unlikely that this state is a product of cold shock - a conclusion reinforced by the fact that we observe no other cold shock associated proteins within the set of upregulated genes. *priB* ($log_2FC = 0.37$, adjusted p-value = 6.4E-12) encodes a protein within the primosome which catalyzes lag strand priming during DNA replication. Conversely, genes involved in pyrimidine biosynthesis (*pyrI, pyrB, pyrC*) were significantly downregulated within the cluster. Interspersed with cluster #0 on the dimensionally reduced plot, we observe a group of cells (cluster #8, 141 cells) highly upregulating genes from the *fim* operon, as discussed in the main text. Cells in cluster #2 appear less active overall, as all genes found to be significantly differentially expressed within the cluster are downregulated compared to the rest of the population. This may be an effect of poor probe penetration or indicative of a more basal cell state. Cluster #3 reveals no significantly overrepresented gene sets by enrichment analysis after accounting for the false discovery rate. Upregulated genes reported in the cluster include *ompT* and *ompX* as well as *tolB* and *tolC* ($log_2FCs > 0.25$, adjusted p-values < 1E-14). In addition, scRNA-seq reveals a cluster of cells (cluster #4, 403 cells, 12% of total) that appear to be entering stationary phase; exhibiting a relative increase in expression of *dps* ($log_2FC = 0.91$, adjusted p-value = 4.17E-26). *dps* encodes a protective protein which binds to the bacterial chromosome and sequesters intracellular Fe_2+, forming a highly stable protein-DNA mineral complex which protects the DNA from oxidative damage. In *E. coli*, *dps* has been associated with the transition into stationary phase as cells quickly work to preserve genetic material from stressors including UV irradiation, metal toxicity, thermal fluctuations, and acid/base shock. Cluster #9 (117 cells, 3.5% of total) was also shown to upregulate dps ($log_2FC = 1.05$, adjusted p-value = 7.04E-16) while highly expressing the *gad* (*gadA, gadB, gadC*) and *hde* (*hdeA, hdeB, hdeD*) operons. Both operons encode resistance to acid stress and fall under the gene set of "regulation of intracellular pH" (fold enrichment > 100, FDR = 6.72E-03). *gadA* and *gadB* produce the subunits of glutamate carboxylase which allows cells to interconvert between L-glutamate and GABA by incorporation of an intracellular proton while *gadC* encodes the antiporter enabling L-glutamate uptake and GABA export. *hdeA* and *hdeB* encode acid stress chaperone proteins with differing pH optimums; both preventing the aggregation of periplasmic proteins denatured under acidic conditions. *osmY*, encoding a periplasmic chaperone protein, was also upregulated within this cluster and has been shown to be induced by entry into the stationary phase. Altogether, DGE analysis reveals a fraction of cells within the population that are experiencing significant stress. Cells in cluster #4 appear to be at the initiation of this state while cells in the cluster #9 are more clearly induced, expressing dps along with proteins dealing with acid, osmotic, and oxidative stresses. It is possible these clusters represent cells transitioning into stationary phase or, alternatively, a transient state experienced during active metabolism/exponential growth. Cells within clusters 5 and 7 upregulate genes involved in pyrimidine biosynthesis as discussed in the main text. Interestingly, cluster #5 differentially overexpresses *argG* ($log_2FC = 0.89$, adjusted p-value = 6.57E-29), although no other genes specific to arginine biosynthesis were found to be upregulated. Both clusters upregulate *ygjF*, encoding a G/U mismatch-specific DNA glycosylase to correct mispairings in double stranded DNA that arise from alkylation or deamination of cytosine. *ygjF* is often active in stationary-phase cells[2]. Cluster 6 is defined by the upregulation of genes involved in arginine biosynthesis as discussed in the main text.

Supplemental note 4: proBac-seq is compatible with non-PCR barcoding of single cell transcripts - as seen in Figure S20 and use of droplet RT instead of PCR

To see if our microfluidic probe-based methods are robust with different DNA-labeling chemistries, we repeated scRNA-seq on *B. subtilis*, replacing in-droplet PCR used to generate data in Figure 2 with a probe barcoding step utilizing a reverse transcriptase enzyme in accordance with the 10X

Chromium Single Cell 3' standard protocol for eukaryotic scRNA-seq. While the approach in Supplementary Figure S20 (RT in drops instead of PCR) proved less sensitive in transcript capture compared to the final, optimized method, we still resolve distinct biological states upon dimensional reduction of expression vectors including cell subpopulations corresponding to competence and sporulation. Additionally, we observe a majority grouping of cells (clusters #0-5) with significantly increased expression of ribosomal genes as well as genes involved in purine and amino acid biosynthesis – corresponding to the "metabolically active" subpopulation defined previously. scRNA-seq also revealed a subpopulation of cells (cluster #8, 1.9% of cells) within the sample exhibiting a state unforeseen in the previous run - characterized by the upregulation of genes encoding a manganese importer (*mntABCD*, $\log_2$FCs > 1.99, adjusted p-values < 1E-11), sigma factor *sigW* ($\log_2$FC = 1.69, adjusted p-value = 5E-16), and stress response proteins (*yuaF, yceDEF, yqfA, yqeZ*, $\log_2$FCs > 0.99, adjusted p-values < 3.3E-04) involved in maintaining cell membrane integrity. *yceF*, specifically, is a cell envelope stress protein that conveys resistance to manganese. katA, an iron-binding catalase which protects cells against toxification from hydrogen peroxide, was also upregulated. Altogether, this cluster of cells appears to be responding to stress induced by a low concentration of manganese within the environment by upregulating manganese uptake machinery. *maeA* was also upregulated in these cells ($\log_2$FC = 1.9, adjusted p-value = 3.65E-34). maeE encodes a malic enzyme necessary for catabolic repression of pyruvate import (pftAB) as well as for maintaining ATP levels during growth on the carbon source by conversion of malate to pyruvate and generation of NADH.

Supplemental note 5: Determination of probe concentrations

To determine the concentration of probes needed to saturate an approximate number of transcripts in solution, saturation was defined as total, nonspecific probe coverage for each transcript. In other words, a pool of transcripts is considered saturated when, for each molecule, there is an entire library of probes (n = 1 probe per unique design) exclusively dedicated. In reality, this system would be over-saturated as it contains enough probes to tag every transcript even if all transcripts have the same sequence.

In our protocol, we prepare 150 uL of fixed cells from a 2X concentrated culture collected at mid-log phase. Assuming the culture contains 1E8 cells/mL prior to concentration and each bacterial cell contains 2,000 transcripts [13], the following estimate can be made using a probe set containing 20,000 unique ssDNA probes of length 138 bp (~44,000 g/mol).

$$\frac{2E8\ cells}{mL}\left(\frac{2000\ transcripts}{cell}\right) = \frac{4E11\ transcripts}{mL} = 0.66\ nM\ transcipts$$

$$\frac{1\ probe\ set}{transcript}\left(\frac{20{,}000\ unique\ probes}{1\ probe\ set}\right) = \frac{20{,}000\ probes}{transcript}$$

$$0.66\ nM\ transcripts\ \left(\frac{20{,}000\ probes}{transcript}\right) = 13{,}020\ nM\ probes = 13.2\ uM\ probes$$

$$13.2\ uM\ probes\ \left(\frac{1M}{10^6\ uM}\right)\left(\frac{44{,}000g}{mol}\right) = 0.58\frac{g}{L} = 580\frac{ng}{uL}$$

In our final experiments, we achieved a probe concentration of 600 ng/uL. To note, our calculations assume equal representation of all probe designs in the applied probe library which is unlikely due to biases introduced during probe synthesis and amplification. However, this

number likely represents a gross overestimate of probe requirements for a few reasons. Cell concentration and, therefore, transcript load was purposefully overestimated by assuming an exponentially growing culture contains 1E8 cells/mL; this number better represents the carrying capacity of *B. subtilis* in minimal media. In addition, our definition of "saturation" was overly strict, as discussed above. Lastly, probe libraries were designed redundantly to minimize noise in the downstream analysis by creating multiple probes to target different regions of the same gene. For the *B. subtilis* library, we designed, on average, 7 probes per gene - further assuring that our final probe concentration is sufficient to tag all available transcripts for scRNA-seq.

Supplemental note 6: Probe design approach and limitations, probe-based signal analysis and ProBac seq protocol optimization

Probe design was based on parameters established for traditional microarray experiments, which have been optimized extensively in the literature. A common feature to several large commercial microarray manufacturers (Agilent, Affymetrix, Illumina) is usage of probes that have a uniform length and similar binding characteristics. The size of probes in such commercial microarrays is typically 50 or 60 bp, allowing multiple probes to be designed for even relatively short sequences. In our designs we used 50bp of sequence for probe binding and either relied on published commercial array designs (*B. subtilis* and *E. coli*) or designed probes using UPS2 (unique probe selector 2, a free online design tool). In the latter case probes were designed not to overlap and to contain unique binding properties using the standard UPS2 design algorithm. As with all probe-hybridization based analyses (microarray, Northern and Southern blotting, FISH etc) there is a level of noise that may occur due to non-specific binding or insufficient washing and removal of unbound probes. In addition, some probes may be poor at target binding, as has been reported for targets with extensive secondary structure. In traditional microarrays and in ProBac-seq the usage of multiple probes per gene-target allows for different bioinformatic methods to analyze signal. In our experiments we find that two straightforward methods, looking at the median probe signal, or looking at the max probe, yield results that provide biologically similar interpretation (see Methods, Supplementary Figure S16 and S17 and Supplementary Table S10). In addition to optimization of probes, in order for probes to target intracellular transcripts the In-Situ Hybridization reactions must be optimized. In our experimental design we first optimized probe penetration and sample integrity by testing the ability of a fluorescent DNA probe targeting rRNA to enter fixed and treated cells without cell lysis. This was done by treating fixed *E. coli* or *B. subtilis* cells with cell wall degrading enzymes and using fluorescently labeled 16S rRNA probe (FAM labeled probe EUB338: 5' GCTGCCTCCCGTAGGAGT 3') to measure in-situ probe hybridization signal. Addition of lysozyme increased the ability of DNA probes to bind transcripts, while adding mutanolysin to the samples caused cell lysis as seen in Supplementary Figure S12. Based on these results we included lysozyme in the *in-situ* hybridization protocols.

Supplemental note 7: Reproducibility of biological replicates

We looked at the reproducibility of samples in three different ways. First, signal produced using ProBac-seq on a bulk sample of *B. subtilis* cells was compared to the ProBac-seq sample provided by aggregating the data from thousands of single cells from a separate sample that were analyzed in the same stage of growth. This data (Figure 1g) shows high gene-gene correlations between these biological replicates at the bulk level. Second, to make a more direct single-cell

comparison of replicates we compared the output produced by single cell analysis of two *C. perfringens* cultures grown separately in BHI medium without the addition of acetate (biological replicates). This data (Supplementary Figure S18) includes UMAP projections of all cells from the combined dataset, demonstrating that cells from both samples clustering overlaps in the same UMAP space. In addition, a scatter plot of mean frequencies of genes from the two replicates is provided as a quantitative measure of sample-to-sample similarity. The mean is computed across all resolved cells and the correlation score is r^2=0.989. We also report in the figure a similar plot showing variance of frequencies with an r^2=0.972. Finally, as a third measure of reproducibility in ProBac-seq samples we have compared the output of ProBac-seq using two different molecular biology strategies for labeling probes with molecular cell ID. In all main figures and data used in the study we rely on in-droplet PCR to label probes but, as demonstrated in Supplementary Figure S20, probes can also be labeled using reverse transcription. We used this scheme to look at single cells from *Bacillus subtilis* grown in the same conditions used in Figure 2 and find that the two techniques are able to resolve similar cell types (see Supplemental Note S4 for more detail).

Supplemental note 8: Use of 10X microfluidic encapsulation with small particles

The ability of the 10X Chromium microfluidic system to encapsulate smaller sized particles has been previously documented. Specifically, the 10X platform has been used to encapsulate small eukaryotic cells including sperm cells[3] and yeast cells[4-6], whose size is closer to that of bacteria (1-10 uM) than to a typical mammalian cell (100 uM). Furthermore, the 10X platform has been successfully used to analyze isolated nuclei including sperm nuclei[7-8] which are approximately the same size as the bacterial cells analyzed in this paper. This, in addition to the barnyard data (Figure 1) and ability to recover distinct, known cell states (sporulation and competence in *B. subtilis* and fimbriae in *E. coli*) in our study, affirms that the 10X microfluidics system flow properties are compatible with small particle encapsulation. We also found that the dead volume remaining in the inlet wells of the 10X microfluidic device at the end of the run remains unchanged when loading bacterial samples and is consistent with 10X's advertised recovery of approximately 60% for mammalian cell encapsulation.

1. Lun, A. T. L. *et al.* EmptyDrops: distinguishing cells from empty droplets in droplet-based single-cell RNA sequencing data. *Genome Biol.* **20**, 63 (2019).
2. Durfee, T., Hansen, A.-M., Zhi, H., Blattner, F. R. & Jin, D. J. Transcription Profiling of the Stringent Response in Escherichia coli. *J. Bacteriol.* **190**, 1084–1096 (2008).
3. Hermann, B. P. et al. The Mammalian Spermatogenesis Single-Cell Transcriptome, from Spermatogonial Stem Cells to Spermatids. Cell Rep. 25, 1650–1667.e8 (2018).
4. Vermeersch, L., Jariani, A., Helsen, J., Heineike, B. M. & Verstrepen, K. J. Single-Cell RNA Sequencing in Yeast Using the 10× Genomics Chromium Device. Methods Mol. Biol. 2477, 3–20 (2022).

5. Jariani, A. et al. A new protocol for single-cell RNA-seq reveals stochastic gene expression during lag phase in budding yeast. Elife 9, e55320 (2020).
6. Jackson, C. A., Castro, D. M., Saldi, G.-A., Bonneau, R. & Gresham, D. Gene regulatory network reconstruction using single-cell RNA sequencing of barcoded genotypes in diverse environments. Elife 9, e51254 (2020).
7. Eraslan, G. et al. Single-nucleus cross-tissue molecular reference maps toward understanding disease gene function. Science 376, eabl4290 (2022).
8. Tan, H. et al. Single-Cell RNA-seq Uncovers Dynamic Processes Orchestrated by RNA-Binding Protein DDX43 in Chromatin Remodeling during Spermiogenesis. bioRxiv 2022.06.12.495783 (2022) doi:10.1101/2022.06.12.495783.
