# Supplementary material for: Probe-based bacterial single-cell RNA sequencing predicts toxin regulation
Source: Nat Microbiol. 2023 Apr 3;8(5):934–45. doi: 10.1038/s41564-023-01348-4 (PMC10159851; doi:10.1038/s41564-023-01348-4)
Supplement: Supplementary file 2 — Reporting Summary [file 41564_2023_1348_MOESM2_ESM.pdf]

nature portfolio

Corresponding author(s): Adam Rosenthal and Sahand Hormoz

Last updated by author(s): Feb 10, 2023

Reporting Summary

Nature Portfolio wishes to improve the reproducibility of the work that we publish. This form provides structure for consistency and transparency in reporting. For further information on Nature Portfolio policies, see our Editorial Policies and the Editorial Policy Checklist.

Statistics

For all statistical analyses, confirm that the following items are present in the figure legend, table legend, main text, or Methods section.

n/a	Confirmed	
☐	☒	The exact sample size (*n*) for each experimental group/condition, given as a discrete number and unit of measurement
☐	☒	A statement on whether measurements were taken from distinct samples or whether the same sample was measured repeatedly
☐	☒	The statistical test(s) used AND whether they are one- or two-sided<br>*Only common tests should be described solely by name; describe more complex techniques in the Methods section.*
☐	☒	A description of all covariates tested
☐	☒	A description of any assumptions or corrections, such as tests of normality and adjustment for multiple comparisons
☐	☒	A full description of the statistical parameters including central tendency (e.g. means) or other basic estimates (e.g. regression coefficient) AND variation (e.g. standard deviation) or associated estimates of uncertainty (e.g. confidence intervals)
☐	☒	For null hypothesis testing, the test statistic (e.g. *F*, *t*, *r*) with confidence intervals, effect sizes, degrees of freedom and *P* value noted<br>*Give P values as exact values whenever suitable.*
☐	☒	For Bayesian analysis, information on the choice of priors and Markov chain Monte Carlo settings
☒	☐	For hierarchical and complex designs, identification of the appropriate level for tests and full reporting of outcomes
☐	☒	Estimates of effect sizes (e.g. Cohen's *d*, Pearson's *r*), indicating how they were calculated

Our web collection on statistics for biologists contains articles on many of the points above.

Software and code

Policy information about availability of computer code

Data collection	Sequence reads were collected on an Illumina Nextseq 2000 with default base calling software.
Data analysis	Custom code written in MATLAB 2019a (Mathworks) and Python (v3.8) was used to filter and reformat sequencing data and single-cell count matrices and is publicly available (https://gitlab.com/hormozlab/bacteria_scrnaseq). CellRanger 3.1.0 (10x Genomics) was used to obtain single-cell count matrices from sequencing data. Seurat v3.1.2 and Scanpy v1.7 packages were used to perform single-cell analyses. The DeSeq2 (v3.16) package was used for bulk differential gene expression analysis. NovoExpress v1.6.1 was used for flow cytometry analysis. Additional software packages used for manipulating/aligning sequence data include samtools v1.8, featureCounts v2.0, and bowtie2 v2.4. Figures were compiled in Adobe Illustrator 2022.

For manuscripts utilizing custom algorithms or software that are central to the research but not yet described in published literature, software must be made available to editors and reviewers. We strongly encourage code deposition in a community repository (e.g. GitHub). See the Nature Portfolio guidelines for submitting code & software for further information.

Data

Policy information about availability of data

All manuscripts must include a data availability statement. This statement should provide the following information, where applicable:

- Accession codes, unique identifiers, or web links for publicly available datasets
- A description of any restrictions on data availability
- For clinical datasets or third party data, please ensure that the statement adheres to our policy

All sequence data used in this publication is publicly available through the NCBI's GEO repository under accession # GSE223752. The genome for Clostridium perfringens has been deposited in NCBI under the accession numbers CP109957-CP109962.

Human research participants

Policy information about studies involving human research participants and Sex and Gender in Research.

Reporting on sex and gender	Not applicable (non-human research only
Population characteristics	Not applicable (non-human research only
Recruitment	Not applicable (non-human research only
Ethics oversight	Not applicable (non-human research only

Note that full information on the approval of the study protocol must also be provided in the manuscript.

Field-specific reporting

Please select the one below that is the best fit for your research. If you are not sure, read the appropriate sections before making your selection.

☒ Life sciences ☐ Behavioural & social sciences ☐ Ecological, evolutionary & environmental sciences

For a reference copy of the document with all sections, see nature.com/documents/nr-reporting-summary-flat.pdf

Life sciences study design

All studies must disclose on these points even when the disclosure is negative.

Sample size	No sample size calculations were performed prior to experimentation. Single cell datasets were carried out with greater than 1,000 cells in each sample in concordance with standard practice for 10X scRNA-seq experiments.
Data exclusions	No data was excluded.
Replication	Replicates for specified single cell experiments are provided in the supplementary information file.
Randomization	No humans or animals were used. Bacterial cultures that were treated or untreated were taken from the same starter culture and distributed evenly into the control or treated groups.
Blinding	No blinding was performed. Single cell transcriptomes were clustered in an unbiased manner via established methods (UMAP, Louvain algorithm) prior to interpretation.

Reporting for specific materials, systems and methods

We require information from authors about some types of materials, experimental systems and methods used in many studies. Here, indicate whether each material, system or method listed is relevant to your study. If you are not sure if a list item applies to your research, read the appropriate section before selecting a response.

Materials & experimental systems

n/a	Involved in the study
☐	☒ Antibodies
☒	☐ Eukaryotic cell lines
☒	☐ Palaeontology and archaeology
☒	☐ Animals and other organisms
☒	☐ Clinical data
☒	☐ Dual use research of concern

Methods

n/a	Involved in the study
☒	☐ ChIP-seq
☐	☒ Flow cytometry
☒	☐ MRI-based neuroimaging

Antibodies

Antibodies used: Custom ordered polyclonal antibodies against C. perfringens toxin NetB - from ProSci (Poway) used at 1:1000

Validation: Western blot with NetB and with C perfringens strains with or without NetB show that the antibodies produced a band at the expected size only at lanes from purified NetB or media from cells that carry the gene

Flow Cytometry

Plots

Confirm that:

☒ The axis labels state the marker and fluorochrome used (e.g. CD4-FITC).

☒ The axis scales are clearly visible. Include numbers along axes only for bottom left plot of group (a 'group' is an analysis of identical markers).

☒ All plots are contour plots with outliers or pseudocolor plots.

☒ A numerical value for number of cells or percentage (with statistics) is provided.

Methodology

Sample preparation: Cells carrying a promoter fusion were analyzed in growth media without fixation or further staining

Instrument: Acea Novocyte Quanteon

Software: Acea Novocyte Quanteon default software (NovoExpress v1.6.1)

Cell population abundance: No sorting was done, only analysis by flow-cytometry. Population sizes are reported on the figure.

Gating strategy: FSC and SSC gating were used - and gating strategy (FSC/SSC gates) are shown in supplementary figures

☒ Tick this box to confirm that a figure exemplifying the gating strategy is provided in the Supplementary Information.
